# A multi-disciplinary approach for uranium exploration using remote sensing and airborne gamma-ray spectrometry data in the Gebel Duwi area, Central Eastern Desert, Egypt

**DOI:** 10.1038/s41598-024-69147-3

**Published:** 2024-08-26

**Authors:** Mahmoud Abd El-Rahman Hegab

**Affiliations:** National Authority for Remote Sensing and Space Sciences, Cairo, Egypt

**Keywords:** Uranium exploration, Gebel Duwi, Multispectral remote sensing, Gamma-ray spectrometry, Solid Earth sciences, Astronomy and astrophysics

## Abstract

Uranium exploration plays a pivotal role in meeting global energy demands and advancing nuclear technology. This study presents a comprehensive approach to uranium exploration in the Gebel Duwi area of the Central Eastern Desert of Egypt, utilizing remote sensing and airborne gamma-ray spectrometric data. Multispectral remote sensing techniques, including Principal Component Analysis (PCA), Minimum Noise Fraction (MNF), and Band Ratioing (BR), are employed to identify lithological units and hydrothermal alteration zones associated with uranium deposition, such as iron oxides, argillic, propylitic, and phyllic alterations. Additionally, airborne gamma-ray spectrometry data provide insights into the spatial distribution of radioelements, including uranium (eU), thorium (eTh), and potassium (K), as well as radioelement ratios (eU/eTh, eU/K, and eTh/K). The uranium migration index map (eU-(eTh/3.5)) and the F-parameter map (K*(eU/eTh)) have been generated to investigate the movement of uranium within various geological zones and characterize anomalous uranium concentrations. Statistical analyses, including mean (X), standard deviation (S), and coefficient of variability (C.V.), are conducted to identify uranium-rich zones. The integration of these datasets enables the generation of a uranium potential map highlighting areas of elevated concentrations indicative of uranium mineralization. Field observations and mineralogical analyses of collected samples validate our findings, confirming the presence of minerals associated with uranium mineralization in mapped high-potential areas. The significance of minerals like Fe-Chlorite, Fe-Mg-Chlorite, ferrihydrite, goethite, calcite, muscovite, dolomite, actinolite, vermiculite, and gypsum in indicating potential uranium mineralization processes underscores the importance of our results.

## Introduction

The Eastern Desert of Egypt boasts a rich geological tapestry shaped by a complex history, yielding a diverse array of mineral deposits across its expansive terrain. Geologically, it forms part of the esteemed Arabian-Nubian Shield, renowned for its extensive metamorphic and igneous rock formations dating back to the Precambrian era^1,2^. Within this shield lie ancient crystalline structures like granites, gneisses, and schists, forged through dynamic tectonic processes like mountain building and volcanic eruptions^3^. During the Neoproterozoic era, the Eastern Desert experienced profound volcanic and plutonic activities, laying the foundation for the emergence of numerous mineral reserves, including coveted resources like gold, copper, and various base metals^4,5^. This intricate geological history underscores the region's significance as a prime destination for mineral exploration and extraction endeavors.

The Eastern Desert of Egypt boasts a diverse array of both metallic and non-metallic minerals, contributing to its significance as a prime location for mineral exploration and extraction^6^. Among the metallic minerals found in abundance are gold, copper, iron ore, manganese, lead, zinc, and uranium. Iron ore, manganese, lead, and zinc further enrich the region's mineral wealth, presenting opportunities for industrial development^7^. Alongside metallic resources, the Eastern Desert also hosts a variety of non-metallic minerals, including phosphate, limestone, gypsum, and kaolin^8^. These non-metallic resources play pivotal roles in sectors such as construction, agriculture, and manufacturing, contributing to the economic vitality of the region. With its diverse mineral portfolio, the Eastern Desert remains a focal point for ongoing exploration efforts aimed at uncovering its vast geological riches^9^.

Uranium occurrences in the Eastern Desert of Egypt are significant due to their potential as a valuable mineral resource. The region's geological composition, characterized by a diverse array of rock formations such as granitoids, metavolcanics, and metasediments, provides favorable conditions for uranium mineralization^10–12^. Uranium deposits in the Eastern Desert are often associated with granitic intrusions and hydrothermal alteration zones^13,14^. These occurrences are typically found in association with other metals such as gold, copper, and Rare Earth Elements (REEs). The presence of uranium in the Eastern Desert underscores the importance of geological surveys and exploration efforts to assess the region's uranium potential comprehensively^15,16^. Moreover, given the increasing global demand for nuclear energy and the strategic significance of uranium as a fuel source, further research and exploration activities in the Eastern Desert could lead to the identification of economically viable uranium deposits.

Remote sensing satellite imagery provides a powerful tool for detecting and mapping hydrothermal alteration zones, thereby aiding mineral exploration^17–21^. Multispectral satellite images like ASTER and Landsat-9 have been widely used; as these sensors are capable of detecting radiation across multiple spectral bands covering a wide range of wavelengths are essential for detecting minerals. This allows for the characterization of mineralogical compositions based on the absorption, reflection, and emission properties of different minerals across different wavelengths^22,23^. Previous studies have shown the effectiveness of ASTER data in mapping alteration mineral zones for uranium exploration in different areas of the Eastern Desert^24–27^. By employing multispectral remote sensing imagery, researchers can analyze the reflectance patterns of different wavelengths to identify these alteration minerals indicative of uranium mineralization^13,14^. By detecting these minerals through their spectral signatures, remote sensing data can help delineate potential uranium exploration targets and guide ground-based exploration efforts more effectively.

Moreover, the integration of multispectral data with airborne geophysical data can enhance the understanding of the geological context and spatial distribution of uranium mineralization^9,21,28^. Airborne gamma-ray spectrometry data plays an important role in uranium exploration as it helps in identifying and mapping areas with high concentrations of radioactive minerals^13,29^; as anomalies or areas with higher radioactivity, can indicate the presence of uranium or other radioactive minerals. So, the correlation between radiometric data and remote sensing data can provide valuable insights into the concentration and distribution of uranium-bearing minerals.

The primary objective of this research is to explore the new spatial distributions of uranium mineralization in the Gebel Duwi area by analyzing alteration zones and locating uranium point anomalies within the rocks. This exploration is facilitated through the integration of remote sensing satellite imagery and airborne gamma-ray spectrometric data analysis. The aim is to identify and map areas within the study area that exhibit characteristics indicative of potential uranium deposits. This innovative methodology allows for the identification and mapping of potential uranium deposits with greater efficiency than traditional exploration methods. Consequently, the research contributes to the advancement of uranium exploration practices, offering novel insights into the geological context and spatial distribution of uranium mineralization in the Gebel Duwi area.

## Study area and geology

**Figure 1:**
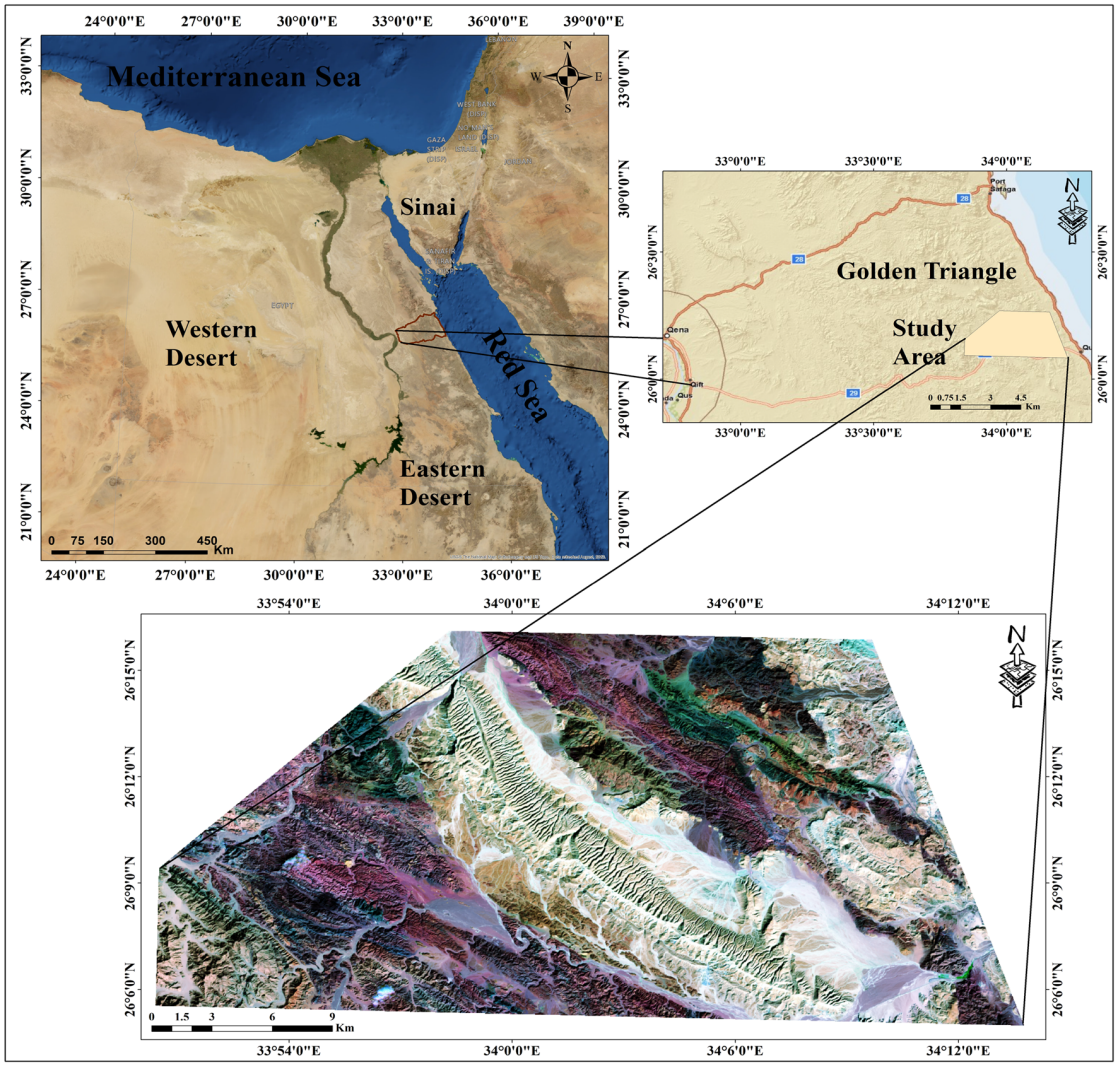
Location map of the Gebel Duwi area, Central Egyptian Eastern Desert. (By ArcGIS v.10.5. https://www.esri.com/en-us/arcgis/products/arcgis-desktop/overview/).

The Gebel Duwi area is located in the Central Eastern Desert of Egypt, positioned on the western side of the Red Sea margin. It lies approximately between latitudes 26°05′ and 26°16′ and longitudes 33°50′ and 34°15′ (Fig. 1).

**Figure 2:**
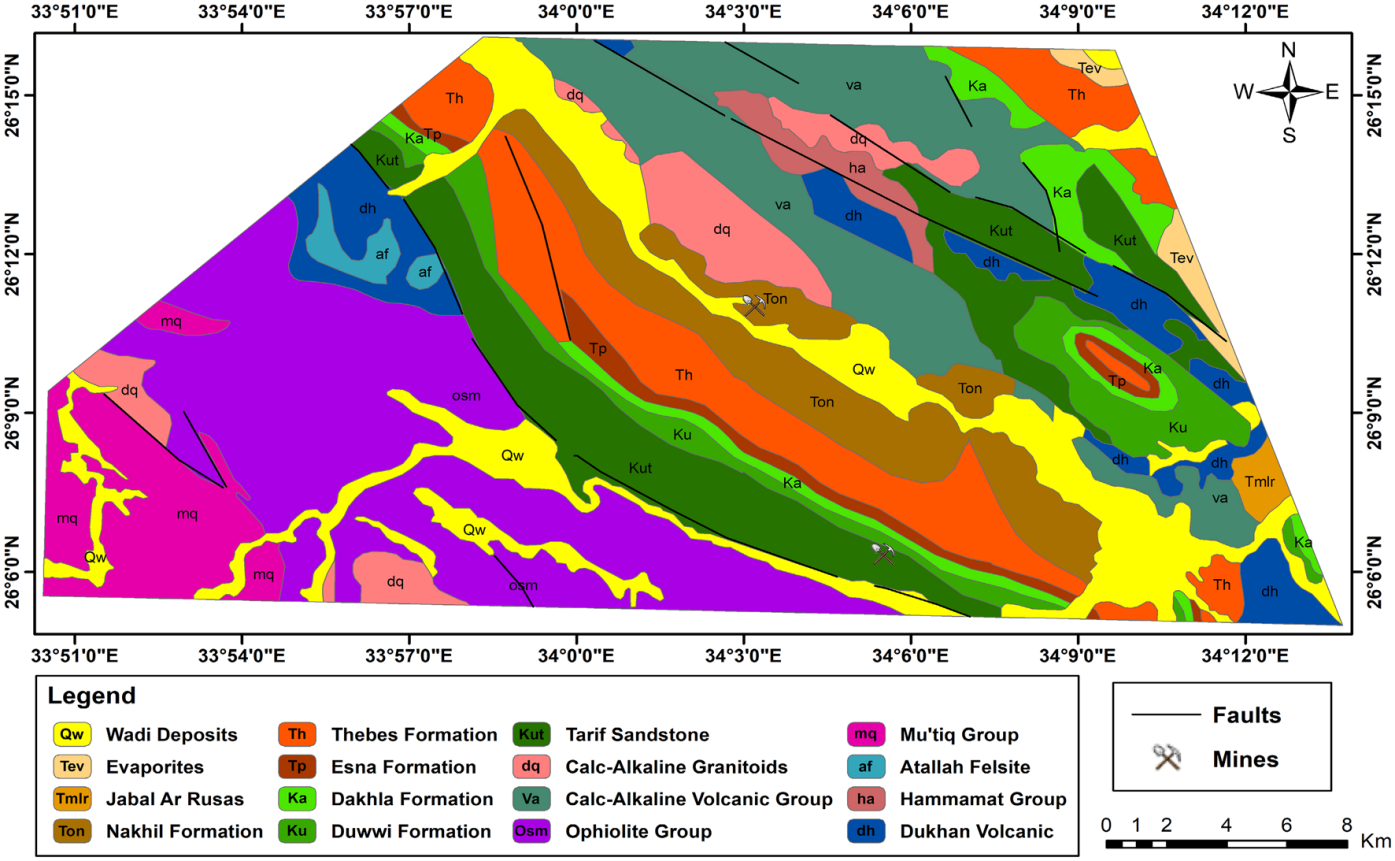
Geological map of the Gebel Duwi area, Central Eastern Desert of Egypt (modified after EGSMA^30^). (By ArcGIS v.10.5. https://www.esri.com/en-us/arcgis/products/arcgis-desktop/overview/).

The geological formations in the study area encompass diverse lithological units and depositional settings. The rock units exposed in the Gebel Duwi area are presented below in chronological order (Fig. 2), with the youngest formations listed as follows: (1) Wadi deposits consist of beach and wadi gravel deposits, alongside silt and sandy bands, including coral reef formations. (2) Evaporites are characterized by steep-sloped gypsum and anhydrite beds, interspersed with green-grey shale and marl intercalations, reaching up to 50 m in thickness. (3) The Jabal Ar Rusas Formation comprises conglomerate, sandstone, and shale layers, occasionally with marl and limestone beds, spanning 110 m thick. (4) The Nakhil Formation features pebbly sandstone, shale with chert clasts, and phosphate-bearing fragments, extending 70 m thick. (5) The Thebes Formation exhibits pale white porcellaneous chalky limestone, with brown/black flint bands and nodules, along with fossiliferous limestone, notably oyster beds. (6) The Esna Formation characterized by yellow-pale brown/green clays and shales rich in foraminifera and nummulites deserti. (7) The Dakhla Formation consists of grey, green, or brown fossiliferous clays, marls, and locally gypsiferous layers, hosting pecten farafrensis fossils. (8) The Duwwi Formation is composed of laminated clays and marls, anhydrite, and limestone, with three phosphate beds (30–100 cm thick). (9) The Tarif sandstone features red-brown, coarse-grained ferruginous sandstone with pebble beds. (10) The Calc-Alkaline Granitoids exhibit coarse grained equigranular, locally k-feldspar megacrystic biotite alkali granite, coarse grained equigranular locally megacrystic monzogranite, coarse grained equigranular variably k-feldspar megacrystic granodiorite. (11) The Calc-Alkaline Volcanic group includes flow breccias, spilitised and pillowform with locally dolerite and microgabbro dykes, aphanitic and plagioclase-phyric andesite, lapilli tuffs, basaltic andesite, massive and laminated porphyritic rhyolite and rhyodacite, rhyolite and dacite, fine-grained laminated to aphanitic cherry (volcaniclastic) ash flow tuffs (volcaniclastic laminae), ironstone (magnetite, haematite) bands. (12) The Ophiolite Group comprises basic lavas, locally pillowform, massive hornblendite gabbro, massive pyroxene-troctolitic gabbro, mesh-textured serpentinite + asbestos + magnesite. (13) The Mut'iq Group consists of metasedimentary and metavolcanic rocks, including medium-grained amphibolite and amphibolitic schist, fine-grained sericite/muscovite, quartz-garnet ± cordierite mylonitic schist, medium-grained quartzofeldspathic metapsammite/felsic mylonite, medium to coarse-grained streaky biotite + hornblende alkali feldspar orthogneiss. (14) The Atallah Felsite comprises pink red brown, fine-grained alkali rich subvolcanic, rare mafic locally rhyolitic. (15) Hammamat Group is composed of polymictic matrix-supported conglomerate, poorly sorted arkosic arenite-greywackes, siltstone and shaly mudstone. (16) Finally, the Dukhan volcanic comprises quartz-feldspar porphyrite subvolcanics, plagioclase-pyroxene andesite, rhyodacite, rhyolite, agglomerate, lapilli tuffs, locally ignimbrite, volcaniclastic conglomerate.

## Datasets and methodology

### Datasets

In this study, various datasets were employed to achieve the research objectives. Landsat-9 imagery was obtained from the U.S. Geological Survey Earth Resources Observation and Science Center (EROS) (https://earthexplorer.usgs.gov/), while ASTER imagery was sourced from NASA Earth Observing System (EOS) (https://www.earthdata.nasa.gov/). Additionally, the Quseir geologic map at a scale of 1:250,000 was acquired from the Egyptian Geological Survey and Mining Authority (EGSMA). Furthermore, airborne gamma-ray spectrometry contour maps, including potassium (K40) contour map (measured in %), equivalent uranium (eU) contour map (measured in ppm), and equivalent thorium (eTh) contour map (measured in ppm), were utilized at a scale of 1:50,000.

#### Remote sensing datasets

Landsat-9, the latest addition to the Landsat satellite series, boasts an array of advanced features tailored for diverse remote sensing applications. Equipped with spectral bands covering Visible (VIS), Near-Infrared (NIR), Shortwave Infrared (SWIR), Panchromatic (PAN), Thermal Infrared (TIR), and Cirrus, Landsat-9 offers versatility in capturing various aspects of Earth’s surface. Its spatial resolutions vary across bands, with 30 m for VIS, NIR, and SWIR, 15 m for PAN, and 100 m for TIR bands 10 and 11. On the other hand, the ASTER sensor provides extensive coverage across Visible Near-Infrared (VNIR), SWIR, and Thermal Infrared (TIR) regions. In VNIR, it encompasses bands 1 (0.52–0.60 μm), 2 (0.63–0.69 μm), and 3N (0.78–0.86 μm), offering detailed insights into surface reflectance characteristics. For SWIR, ASTER includes six bands (bands 4–9) spanning from 1.60 to 2.430 μm, while TIR comprises five bands (bands 10–14) ranging from 8.125 to 11.65 μm. The spatial resolution varies across spectral regions, with VNIR at 15 m, SWIR at 30 m, and TIR at 90 m.

This study utilizes the VIS-NIR-SWIR bands of Landsat-9 to enhance lithological mapping, leveraging their heightened sensitivity to changes in surface reflectance for distinguishing between different rock types^31,32^. Additionally, the VNIR-SWIR bands of ASTER data are employed due to their broad spectral range, typically spanning from approximately 0.52–2.430 μm. These bands enable the detection and identification of various minerals based on their distinctive spectral signatures, while also proving effective in mapping hydrothermal alteration zones associated with mineralization^32,33^.

#### Airborne gamma-ray spectrometry data

The study area was included in an extensive airborne survey conducted by the Aero-Service Division of the Western Geophysical Company of America, spanning across significant portions of Egypt’s central and south Eastern Desert. Conducted in 1984, the primary aim of this project was to identify and assess the mineral, petroleum, and groundwater resources within the region. The airborne gamma-ray spectrometric survey entailed parallel traverse flight lines oriented northeast-southwest (azimuth of 45° and 225° from true north), spaced at 1.5 km intervals. Tie lines were established in a northwest-southeast direction (azimuth of 135° and 315°) with intervals of 10 km. Flying operations maintained an average terrain clearance of 120 m, with aircraft speeds ranging between 220 and 315 km/h. The collected spectral gamma-ray measurements underwent correction, compilation, and presentation as contour maps. Topographic maps and photomosaic base maps at a scale of 1:50,000 were utilized for the survey. The measurements were performed using the Hisens AGRS 3000F, a high-sensitivity 256-channel gamma-ray spectrometer system deployed by Aero-Service for this purpose.

Gamma-ray spectrometry is a powerful tool utilized in uranium exploration due to its capacity to detect and measure gamma radiation emitted by naturally occurring radioactive isotopes, including uranium and its decay products^13,16,34,35^. By analyzing gamma-ray emissions' intensity and distribution within geological formations, gamma-ray spectrometry offers valuable insights into the presence and concentration of uranium-bearing minerals^35–37^. Uranium-rich deposits commonly display heightened gamma-ray counts compared to surrounding non-mineralized rocks, facilitating the identification and delineation of potential uranium ore bodies and exploration targets^13,35,38^.

### Methodology

#### Remote sensing data processing

**Figure 3:**
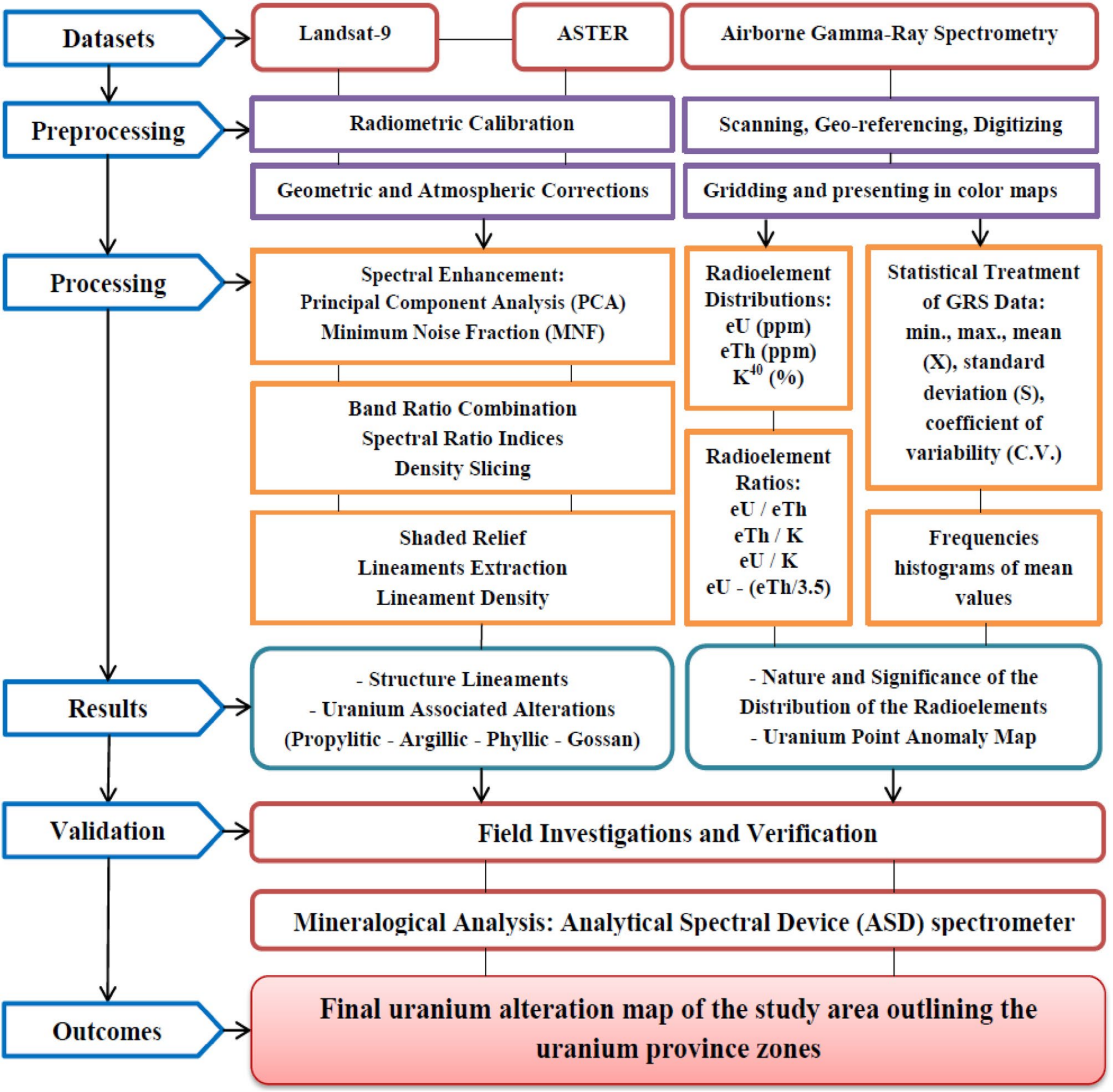
The methodology flowchart.

The logical framework of the methodology adopted in this research is shown in Fig. 3. The satellite images underwent various radiometric and atmospheric calibrations aimed at improving the quality of the raw data and preparing them for subsequent analysis and processing techniques. ENVI 5.3 and Arc GIS 10.5 were used to manipulate, preprocess, and process this data^39^. Initially, FLAASH atmospheric correction was applied to mitigate atmospheric interference and convert the top of atmosphere radiance into reflectance. Subsequently, VNIR and SWIR bands were stacked, and wavelength definitions were established for each band. Following the initial processing, spectral enhancement techniques such as Principal Component Analysis (PCA) and Minimum Noise Fraction (MNF) were implemented on Landsat-9 imagery to differentiate between various rock types. These methods provide valuable insights into the main lithological units^40–42^.

Moreover, the utilization of ASTER data for mapping uranium-related hydrothermal alteration minerals through the Band Ratio (BR) technique has been paramount. This method involves dividing the digital number (DN) values of one band by those of another, proving invaluable for enhancing specific features that may not be discernible in the raw bands alone^43,44^. Additionally, this technique effectively enhances compositional variation by minimizing differences resulting from albedo and topographic slope effects, accentuating the spectral characteristics of rocks and minerals^45^. Therefore, the BR method proves effective for extracting information on hydrothermal alteration minerals^43,45^. Notably, ASTER band ratios such as 4/5, 4/6, and 4/7 in RGB are utilized for highlighting hydrothermal alterations as bright colors^14^, while ratios highlighted in Table 1, such as B3/B1, B4/B2, B5/B8, B4/B7, B4/B6, and B5/B6 are instrumental in delineating hydrothermal alterations associated with uranium deposits, including iron oxides, argillic, propylitic, and phyllic alterations^10–12^.

**Table 1:** The mineral indices adopted through the BR technique.

Mineral indices	References
Fe-Oxides: (B3/B1)	^42^
Gossan: (B4/B2)	^44,46^
Chlorite-Epidote-Carbonates: (B5/B8)	^47^
Kaolinite: (B4/B7)	^42,48^
Sericite: (B4/B6)	^42,44,48^
Muscovite: (B5/B6)	^46^

Subsequently, the application of the density slicing technique on ratio images further refines the results by accentuating various alteration minerals within the study area. This well-established method segments grayscale values ranging from 0 to 255 in single-band images into intervals or slices, each assigned a distinct color. Through this process, the results are refined, and extraneous information is effectively filtered out.

Finally, automated lineaments have been extracted to map linear features, thereby enhancing our understanding of the structural framework within the study area. This approach provides valuable insights into the distribution of hydrothermal alteration features, offering key information about potential mineralized zones.

#### Airborne gamma-ray spectrometry data processing

The airborne gamma-ray spectrometric data underwent processing utilizing the OASIS Montaj mapping and processing system^49^. Initially, the data were acquired and presented as contour maps. Subsequently, the area of interest (Gebel Duwi area) was delineated, digitized, and gridded, and then subjected to qualitative and quantitative interpretations. The maps illustrate the distribution of radioelements (K, eU, eTh), as well as radioelement ratios (eU/eTh, eU/K, eTh/K), alongside the uranium migration index map (eU-(eTh/3.5)) and the F-parameter map (K*(eU/eTh)) have been generated. Through qualitative interpretation, radiation signatures associated with different elements or minerals are identified, facilitating the recognition of potential mineralization and enhancing the overall understanding of the study area.

The quantitative interpretation of the aero-spectrometric data relies on the understanding that both the absolute and relative concentrations of radioelements (K, eU, and eTh) exhibit measurable and significant variations corresponding to different lithological units. Statistical analyses were performed on the airborne spectrometric data in the study area in terms of individual rock types. Key statistical parameters calculated include Maximum (Max), Minimum (Min), Arithmetic Mean (X), Standard Deviation (S), Coefficient of Variability (C.V. = S/X * 100), and specific levels of probability (X + 1S, X + 2S, X + 3S, X−1S, X−2S, and X−3S). Subsequently, potential uranium-rich zones are characterized by heightened uranium concentrations in rocks and soils^13,50,51^. These zones can be identified through a probabilistic approach based on the deviation of the data from the mean background, as delineated by the dataset itself. These deviations are quantified in terms of specific levels of probability. The threshold for identifying high anomalous values is set at two or three standard deviations above the calculated arithmetic mean value (X + 2S or 3S) for each rock unit. Consequently, the uranium point anomaly map is generated, which highlights areas surpassing the thresholds of (X + 2S) and (X + 3S) for the eU, eU/K, and eU/Th variables.

#### Integration and validation

After identifying uranium-related hydrothermal alteration minerals' spatial distribution and correlating them with areas of high-density lineaments and elevated uranium anomalies from qualitative and quantitative analysis of gamma-ray spectrometric data, uranium-rich zones were detected. To validate these findings, a field study was conducted. Seventeen rock samples were extracted from potentially uranium mineralized locations and analyzed using the ASD TerraSpec Halo Mineral Identifier Device for identifying their mineral compositions and their spectral signatures. The TerraSpec Halo Mineral Identifier Device is a cutting-edge tool utilized for the precise identification of mineral composition in various rock samples. Leveraging advanced spectroscopy technology, this device analyzes the unique spectral signatures emitted by minerals when exposed to light. By measuring the reflected wavelengths across a wide spectral range, typically from 350 to 2500 nm, the TerraSpec Halo can accurately identify and quantify the presence of different minerals within a sample^26,52,53^.

## Results and discussion

The Gebel Duwi area holds significant importance in various research domains, making it a focal point for scientific inquiry. Firstly, studies by Dabous ^54^ have highlighted the geological composition of the study area, emphasizing the presence of phosphorite-bearing strata within the Duwi formation. Dabous found that the phosphatic strata within the Duwi formation are tilted, and in certain locations, the ores come into contact with groundwater below the present-day water table. The concentration of uranium (U) in the groundwater in the Red Sea area varies significantly, ranging from 0.024 to 22 ppb, while consistently exhibiting a high 234U/238U activity ratio^54^. Secondly, research by El Ayyat^55^ delves into the paleoenvironmental conditions during the late Cretaceous period, revealing the presence of peloids in phosphorites and limestones within the Duwi formation. This provides valuable insights into the ancient marine ecosystems and the processes involved in sedimentary rock formation^55^. Furthermore, investigations by ElKammar ^56^ suggest the potential of the Dakhla formation within the Gebel Duwi area as an energy resource, particularly due to its composition of organic-rich calcareous shale and argillaceous limestone. This highlights the area’s significance in the exploration of unconventional fuel resources^56^. Abou El-Anwar et al.^57^ conducted a comprehensive analysis of the Duwi formation, identifying phosphate rocks enriched with apatite, calcite, quartz, and trace elements such as uranium (U), thorium (Th), cadmium (Cd), arsenic (As), antimony (Sb), vanadium (V), chromium (Cr), zinc (Zn), copper (Cu), nickel (Ni), and rare earth elements (REEs). This research contributes to our understanding of mineralogy and resource potential in the study area^57^. Moreover, studies by Yousif et al.^58^ utilized GIS and remote sensing techniques to map the paleodrainage network in the Gebel Duwi area, providing valuable information on past, present, and future water resources. That research aids in water resource management and environmental conservation efforts^58^. Additionally, Abou El-Anwar et al.^59^ investigated the geochemistry, mineralogy, and depositional environment of black shales within the Duwi formation. Their research revealed that the formation comprises interbedded shales, marls, reefal limestone, and phosphate lenticular bands. In terms of mineralogy, it contains a variety of minerals including montmorillonite, kaolinite, calcite, gypsum, quartz, and pyrite^59^. Lastly, analyses by Abdelhady et al.^60^ focused on sedimentary analysis to understand the influence of environmental conditions on paleoecosystems. Their research underscores the economic significance of the Duwi formation within the Middle East phosphorite belt, emphasizing its potential as a valuable geological resource as it comprises three distinct facies: organic carbon-rich shales, phosphatized pack/grainstones, and biofloat/rudstones^60^. So, the integration of advanced remote sensing techniques and airborne gamma-ray spectrometric data analysis holds significant promise for investigating the study area to identify potential uranium-rich zones with greater accuracy. This innovative approach represents a significant departure from conventional methods, offering a deeper understanding of the area’s geological composition and uranium distribution.

### Remote sensing data analysis

#### Spectral enhancement for highlighting lithology

##### Principal component analysis (PCA)

The principal component transformation stands as a cornerstone in image processing for geological mapping, widely recognized for its efficacy in converting correlated spectral bands into a concise set of uncorrelated ones termed principal components (PCs)^42,61^. Particularly in multispectral remote-sensing images, this technique, known as PCA, illuminates spectral responses linked to specific minerals resulting from hydrothermal alteration processes^62^.

**Figure 4:**
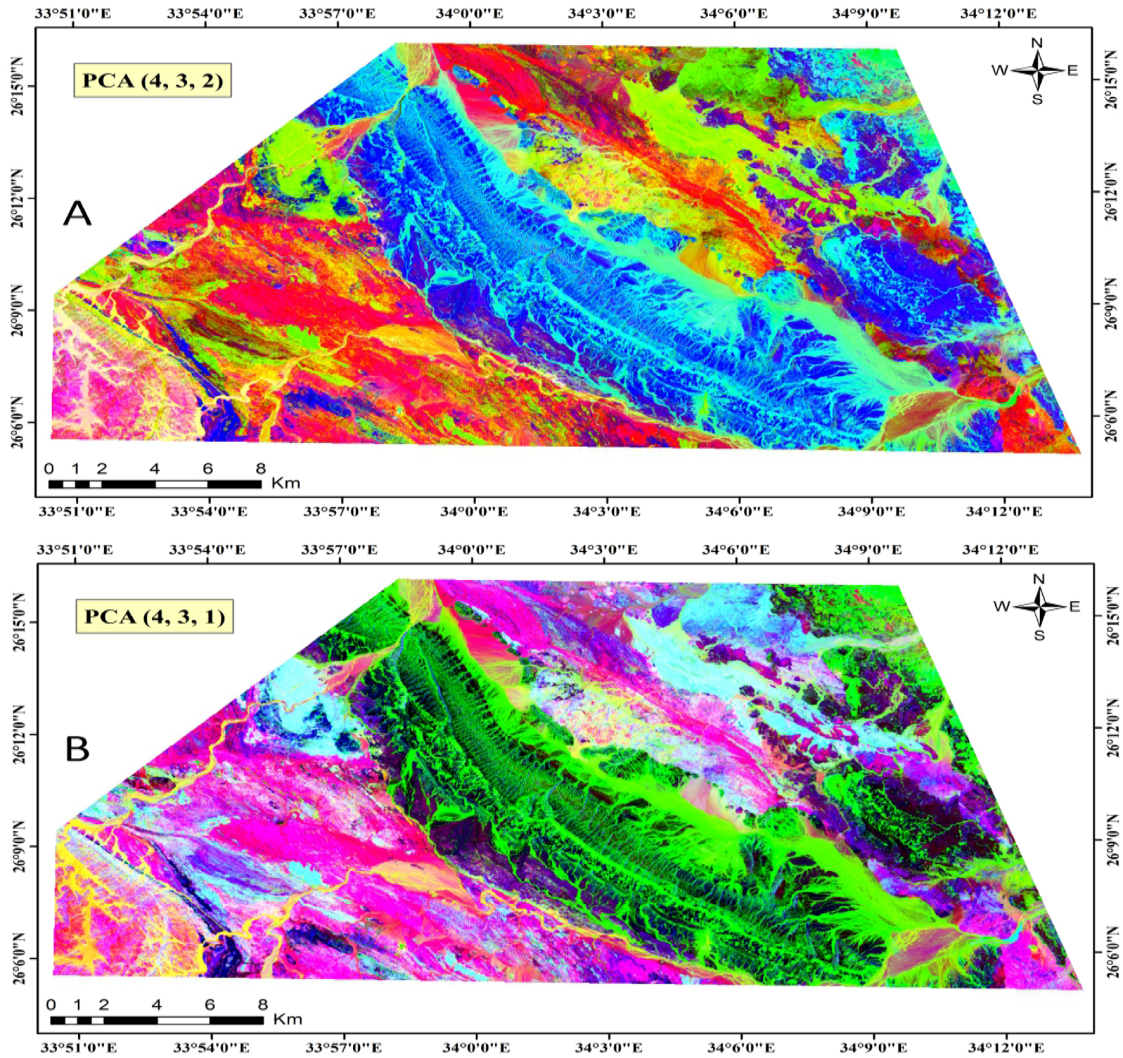
Landsat-9 (PC4-PC3-PC2) and (PC4-PC3-PC1) in RGB respectively. (By ENVI https://www.l3harrisgeospatial.com/Software-Technology/ENVI).

In our investigation, we computed PCs for the seven Landsat-9 VNIR-SWIR bands and generated composite images using PC4, PC3, and PC2, as well as PC4, PC3, and PC1 to represent the red, green, and blue channels, respectively (Fig. 4). Moreover, Table 2 depicts the PCs obtained through PCA on seven bands, presenting coefficients for each band in the corresponding PC. This table furnishes details on eigenvectors, eigenvalues, and the percentage of total variance explained by each PC. Notably, PC1, with the highest eigenvalue and percentage, encapsulates the most significant variance in the data, followed by subsequent components in descending order of importance. By unraveling each band's contribution to the dataset variance, PCA facilitates the identification of the most influential components, thereby proving to be an invaluable tool in distinguishing the lithological units within the study area.

**Table 2:** Eigenvector matrix and eigenvalues of principal component analysis on Landsat-9.

	PC 1	PC 2	PC 3	PC 4	PC 5	PC 6	PC 7
Band 1	− 0.941289	− 0.320656	− 0.064588	− 0.081464	0.006406	− 0.017515	− 0.000149
Band 2	0.336267	− 0.891295	− 0.206277	− 0.213256	0.062889	0.021338	− 0.008947
Band 3	0.010869	− 0.174033	0.951137	− 0.212607	− 0.136197	0.033614	0.007194
Band 4	0.002565	− 0.258969	0.095003	0.882602	− 0.380302	0.008596	0.014593
Band 5	− 0.007046	− 0.073299	0.197633	0.338653	0.877381	− 0.261992	− 0.048954
Band 6	0.025134	− 0.000371	− 0.022084	− 0.092123	− 0.250298	− 0.923219	− 0.274609
Band 7	0.009696	− 0.006958	− 0.006742	− 0.022904	− 0.019466	− 0.277597	0.960129
Eigenvector	0.091303	0.001909	0.000599	0.000183	0.000045	0.000008	0.000002
Eigenvalue %	97.0802454	2.02979298	0.63690204	0.194579421	0.047847399	0.008506204	0.002126551

##### Minimum Noise Fraction (MNF)

**Figure 5:**
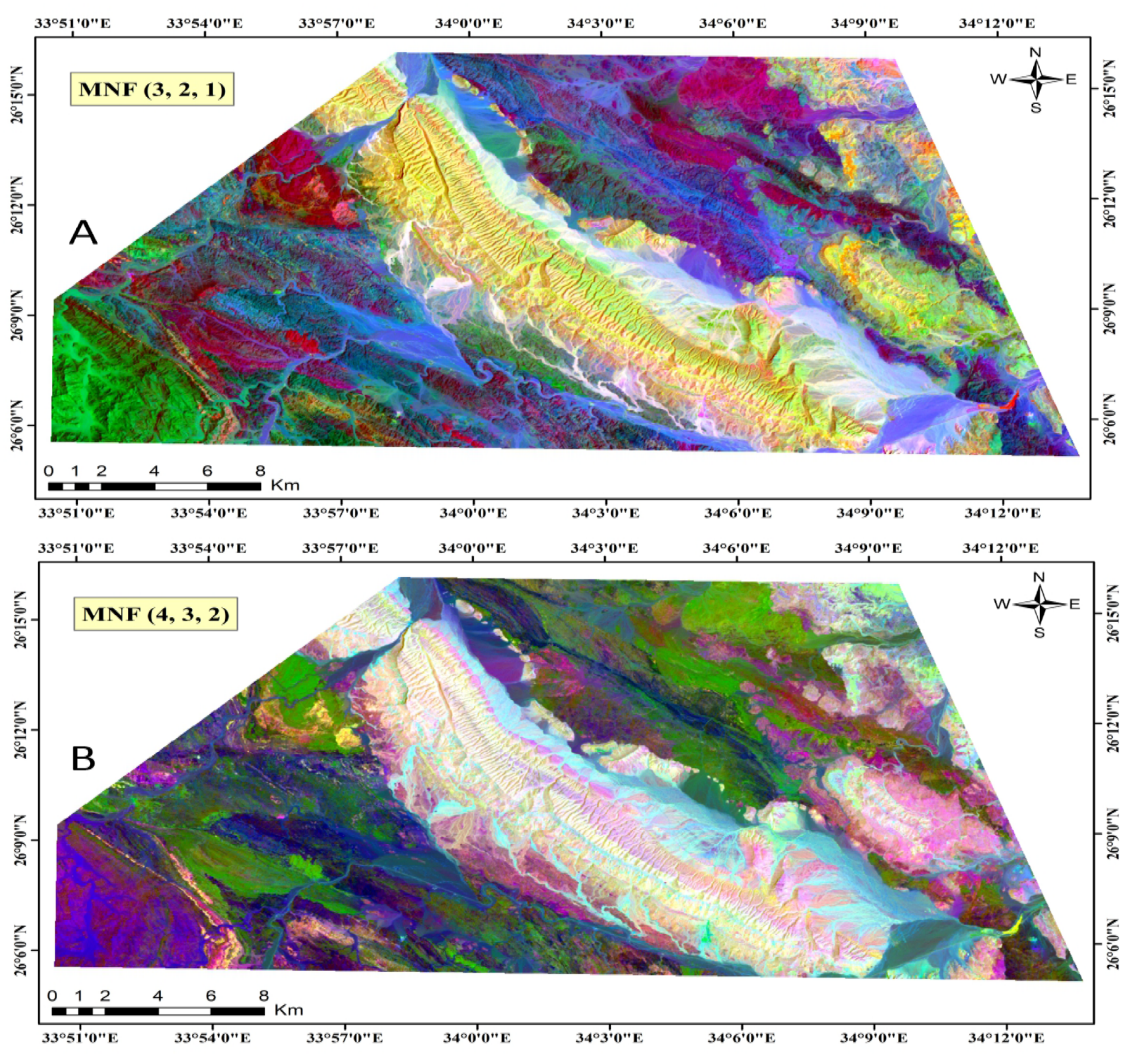
Landsat-9 MNF images, utilizing band combinations (3-2-1) and (4-3-2) in RGB, respectively. (By ENVI https://www.l3harrisgeospatial.com/Software-Technology/ENVI).

The MNF technique^40^ is a sophisticated image processing method widely employed in remote sensing. It works by transforming original multispectral image bands into a new set of components, optimizing the signal-to-noise ratio to enhance relevant information while minimizing noise interference^63^. MNF has proven instrumental in improving the interpretability of remote sensing data and facilitating feature discrimination, particularly in geological mapping applications. Illustrated in Fig. 5 are MNF images generated using MNF components (3, 2, and 1) and (4, 3, and 2), effectively delineating the main lithological units.

#### Mapping uranium-related hydrothermal alterations

**Figure 6:**
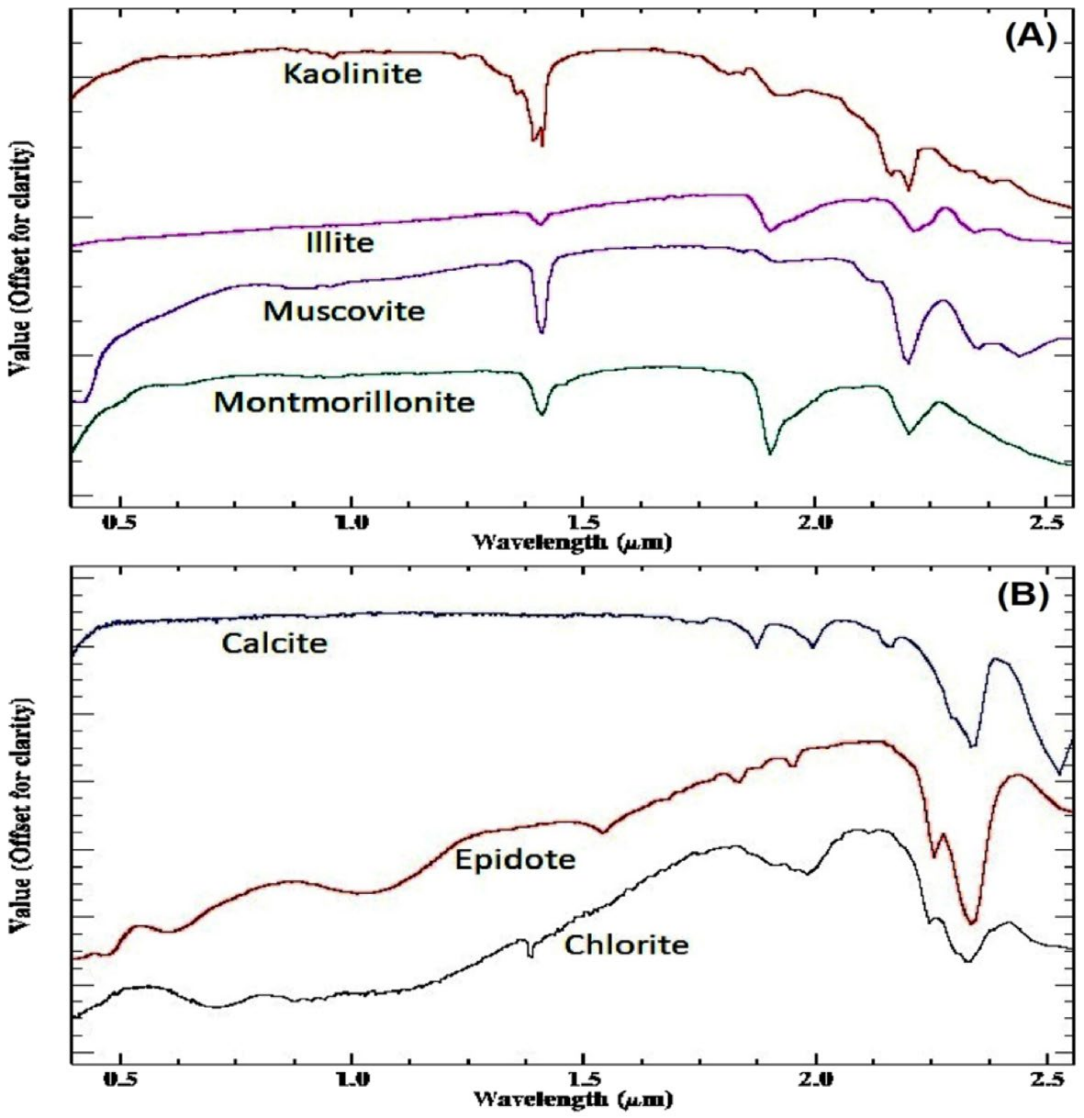
(**A**) A USGS standard mineral spectral curves representing phyllic and argillic alteration zones; (**B**) USGS standard mineral spectral curves representing propylitic alteration zone.

Indeed, alterations related to uranium mineralization frequently manifest as distinct hydrothermal alteration zones, marked by significant changes in mineral composition and structure induced by the presence of hot, mineral-rich fluids. Among the common hydrothermal alteration zones closely associated with uranium deposits are iron oxides, argillic, propylitic, and phyllic alterations^10–12^. The examination of USGS standard mineral spectral curves (Fig. 6) concerning ASTER bands reveals significant insights into the spectral characteristics of various alteration zones. Specifically, Al–OH minerals like kaolinite, muscovite, and montmorillonite, prevalent in argillic alteration zones, demonstrate pronounced reflectance in band 4 of the SWIR region^64^. In contrast, the phyllic zone, dominated by muscovite (sericite), exhibits a distinct Al–OH absorption feature primarily centered at 2.20 μm (ASTER band 6), complemented by a secondary feature near 2.38 μm (ASTER band 8). Reflectance spectra within the propylitic zone unveil notable Fe, Mg-OH absorption features, and CO_3_ features attributed to molecular vibrations in epidote, chlorite, and carbonate minerals, distinctly observed in the 2.35 μm (ASTER band 8) region^65^.

Furthermore, to improve the detection of phyllic alterations characterized by minerals like sericite and muscovite, as well as argillic or advanced argillic alterations containing minerals such as kaolinite and illite, and propylitic alterations involving chlorite, epidote, and carbonate minerals, along with iron oxides like goethite, hematite, and limonite, various band ratios were utilized: band 4/2 for gossan/ iron oxides^44,46^, band 4/6 to highlight the aluminum hydroxide (Al-O-H)^44,48^, band 4/7 for identifying alteration minerals with Al-OH and Fe-OH^48^, band 5/8 for chlorite-epidote-carbonates^47^, band 5/6 for muscovite^46^, band 3/1 for Fe-oxides^42^. Iron oxide minerals are primarily identified through spectral bands 1 and 3, as highlighted in previous studies^42^. These minerals exhibit distinctive absorption features in band 1 and 2, along with reflectance features in band 4^22,25^.

**Figure 7:**
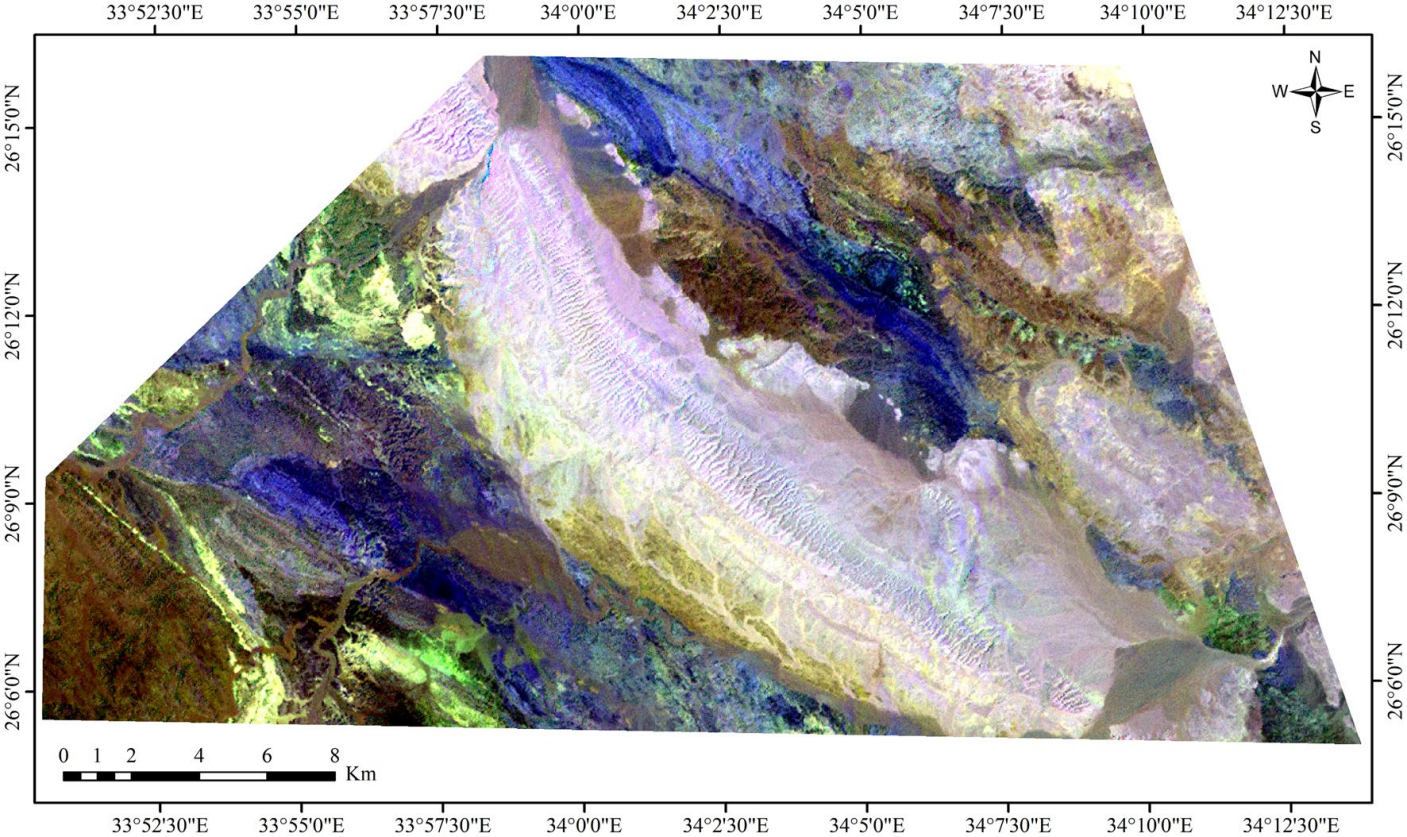
ASTER band ratio image 4/5, 4/6, 4/7 in RGB, showing hydrothermal alterations as bright colors, at the Gebel Duwi area. (By ENVI https://www.l3harrisgeospatial.com/Software-Technology/ENVI).

**Figure 8:**
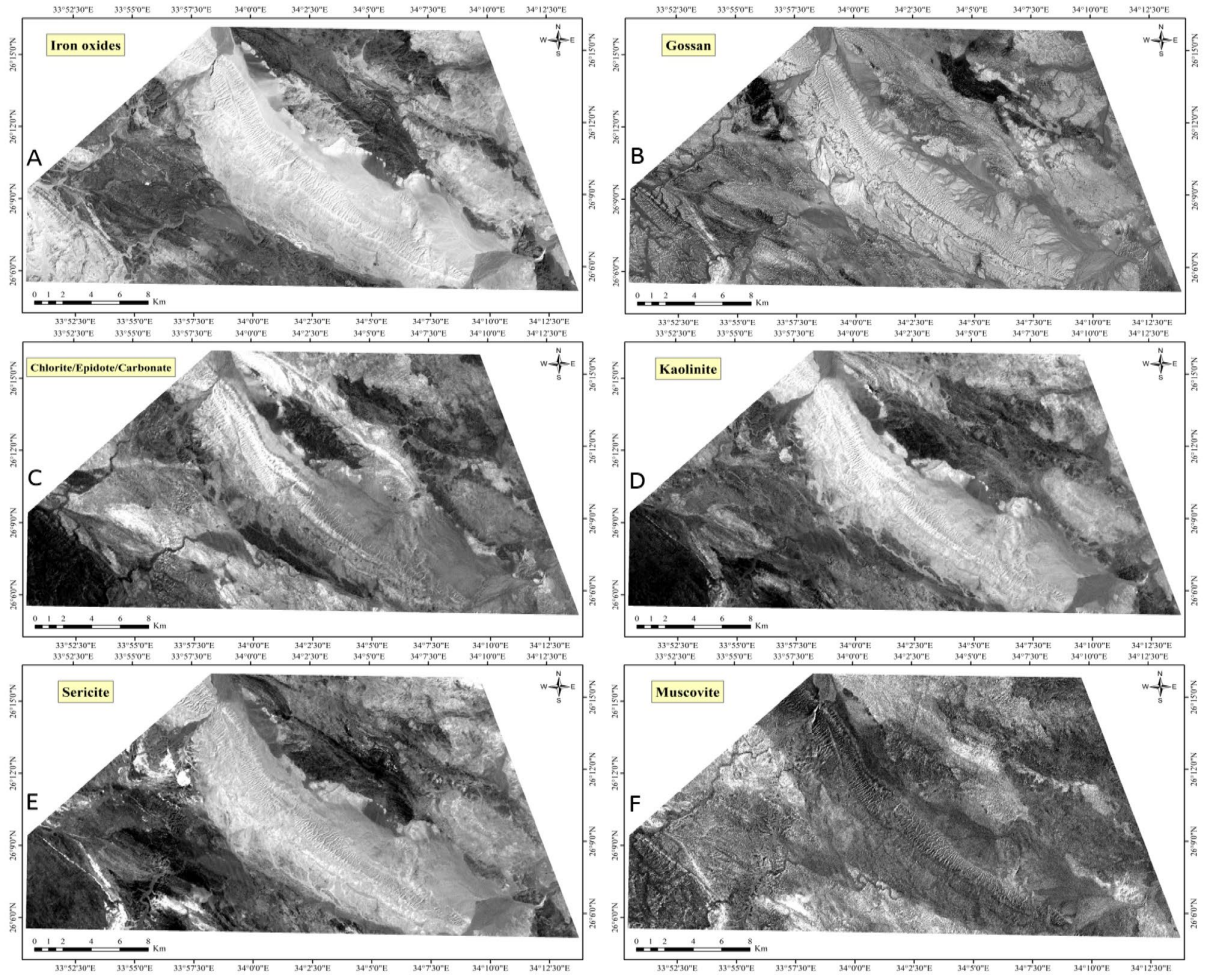
ASTER band ratio images: Iron oxides (B3/B1), Gossan (B4/B2), Chlorite/Epidote/Carbonate (B5/B8), Kaolinite (B4/B7), Sericite (B4/B6), and Muscovite (B5/B6). (By ENVI https://www.l3harrisgeospatial.com/Software-Technology/ENVI).

**Figure 9:**
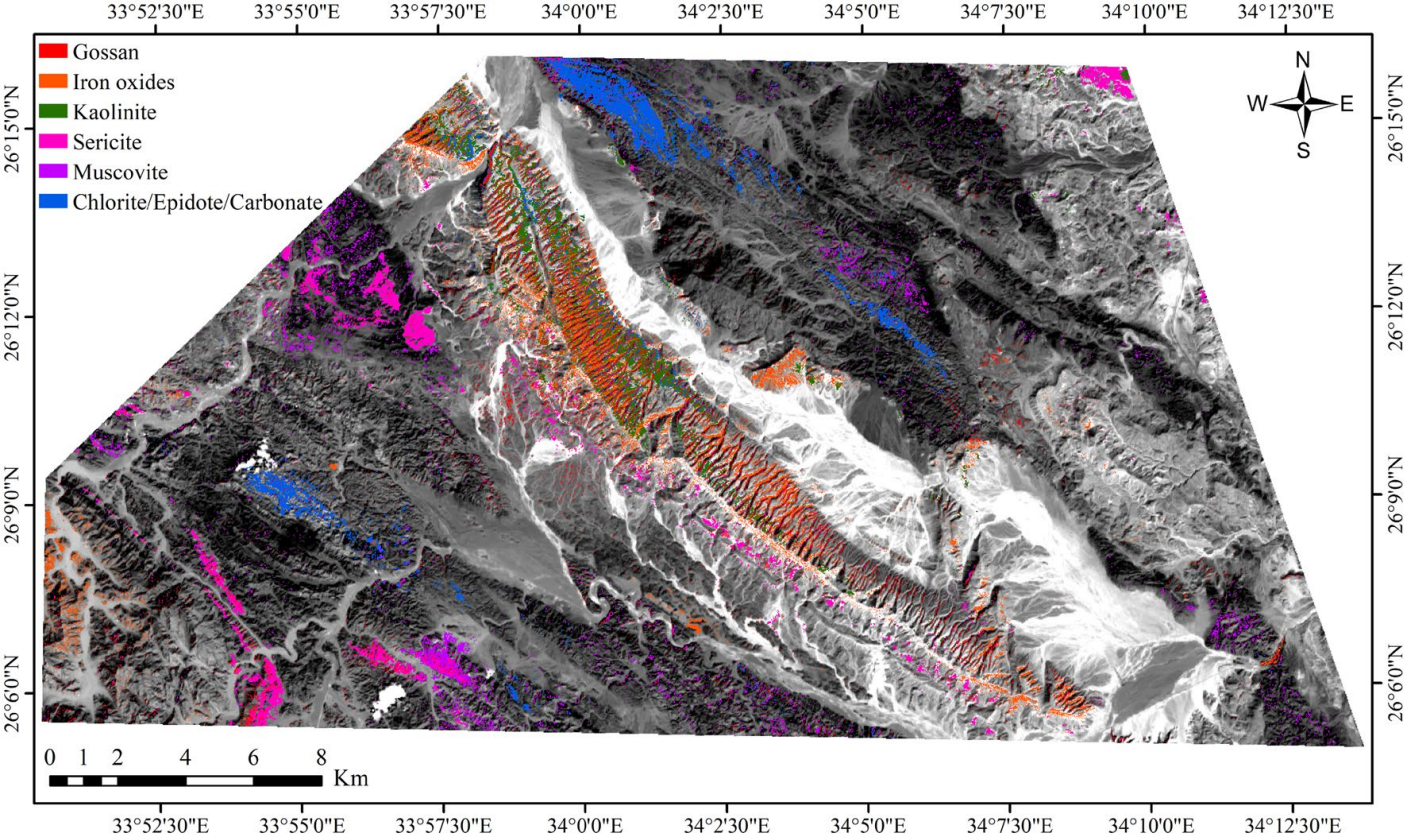
Uranium-related hydrothermal alteration minerals overlaid on ASTER Band 1. (By ENVI https://www.l3harrisgeospatial.com/Software-Technology/ENVI).

Additionally, ASTER ratio images, such as those obtained from bands 4/5, 4/6, and 4/7, are utilized to accentuate hydrothermal alterations (Fig. 7), which are depicted as bright colors in the imagery^14^. The ASTER band ratio 4/6 serves as a reliable indicator of hydrothermal alteration, particularly enhancing the detection of kaolinite and muscovite, which are hydroxyl-bearing minerals, as shown in Fig. 8 and 9. This is attributed to their distinctive spectral signatures, with high reflectance observed in band 4 and lower reflectance in band 6^42^. Additionally, these minerals exhibit an absorption feature in band 6, resulting in high reflectance in bands 4 and 7, and comparatively lower reflectance in band 6^42,66^.

Consequently, ASTER band ratios 4/6 and 4/7 emerge as valuable tools for identifying argillic and phyllic alteration zones^42^. Calcite, characterized by a strong absorption feature in ASTER band 8 and a weak peak in band 5, can also be effectively detected using these spectral ratios^66,67^. Moreover, minerals containing magnesium hydroxide and carbonates, such as chlorite, epidote, and carbonate, exhibit distinct spectral responses in band 8, further enhancing the capabilities of ASTER data in mineral detection and characterization^42^.

#### Mapping surface linear structures

The presence and characterization of lineaments significantly influence mineral exploration endeavors, serving as vital indicators of subsurface geological structures and potential mineral deposits^68^. These linear features comprising faults, fractures, and joints, serve as pathways for mineral-rich fluids, enabling their migration and subsequent deposition^69^. Through mapping and analysis of lineaments, we can pinpoint zones of structural weakness and fluid flow pathways conducive to mineralization. This approach allows us to pinpoint favorable locations for ore deposits^32^.

**Figure 10:**
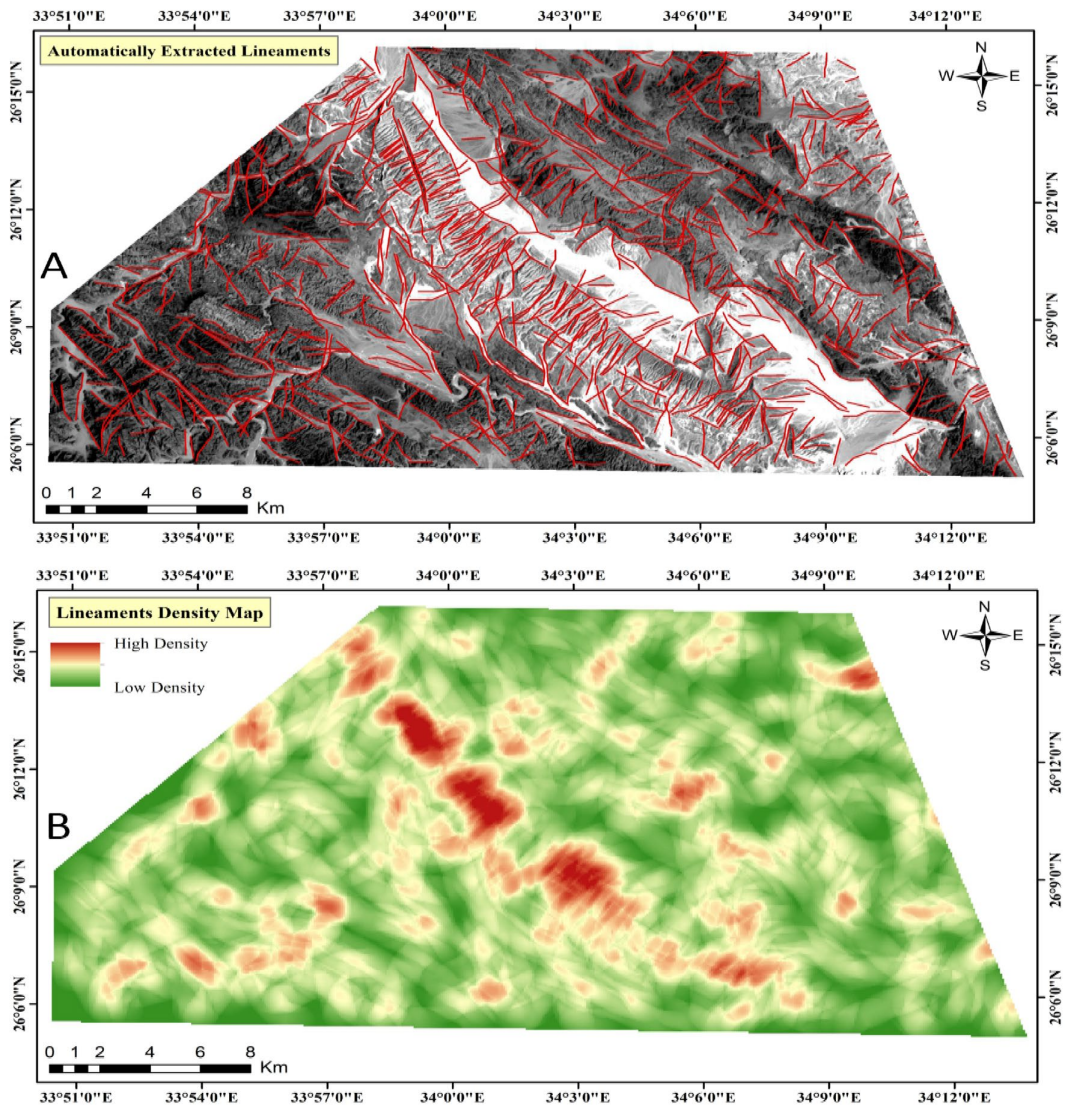
Automatically extracted lineaments from the ASTER image, and lineament density map. (By ArcGIS v.10.5. https://www.esri.com/en-us/arcgis/products/arcgis-desktop/overview/).

The lineaments were automatically mapped (Fig. 10-A) using the “LINE” module within the “PCI Geomatica” software, employing a shaded relief image derived from PCA data covering the study area. A lineament density map was generated (Fig. 10-B), where regions exhibiting high lineament density are considered promising sites for abundant faults and fractures. These structural features serve as conduits for subsurface fluids, including hydrothermal solutions, facilitating their migration and thereby influencing the formation of hydrothermally altered zones^63^.

### Airborne gamma-ray spectrometric data interpretation

#### Qualitative interpretation

##### Description of the radioelements distribution

**Figure 11:**
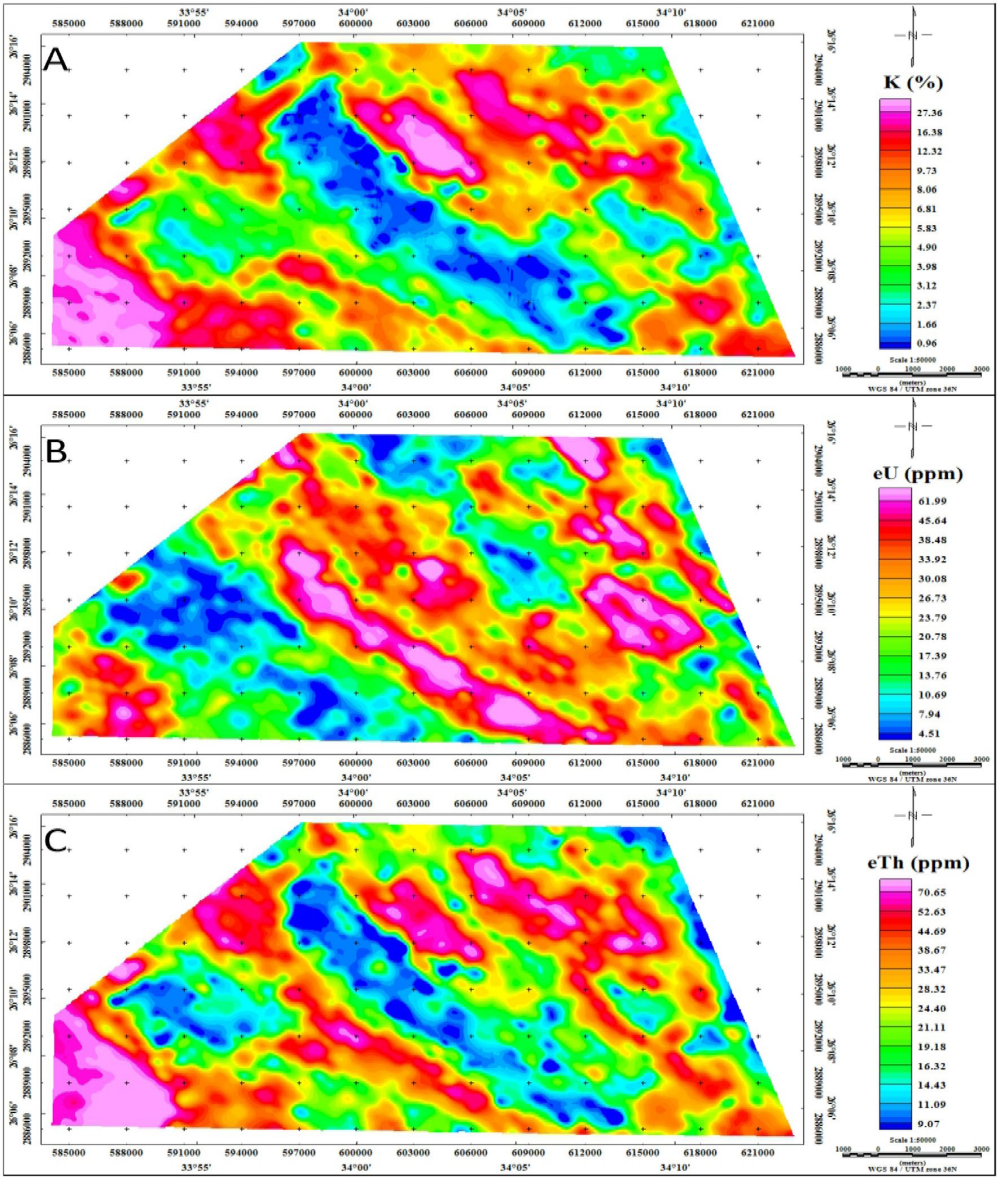
Spatial distribution of potassium radiation (%), equivalent uranium radiation (ppm), and equivalent thorium radiation (ppm) concentrations of the Gebel Duwi area, (Map values*10). (By Geosoft Oasis Montaj software https://www.seequent.com/products-solutions/geosoft-oasis-montaj/).

The airborne gamma-ray spectrometry survey conducted in the study area provided valuable insights into three key variables: potassium concentration (K40, in %), equivalent uranium content (eU, in ppm), and equivalent thorium levels (eTh, in ppm). These variables were mapped to create contour maps, as illustrated in Fig. 11. These maps serve to illustrate the spatial distribution of these radiometric elements, highlighting the variability of different rock and soil types in terms of their surface elemental concentrations^70^. The figure presented in this study offers a comprehensive visualization of the elemental composition across the Gebel Duwi area. The radiometric elements in the study area display a varied distribution across three discernible levels: the first level, characterized by colors ranging from bright magenta to heavy magenta, denotes areas with the highest concentrations; the second level, delineated by colors from bright green to yellow, signifies regions with intermediate values; and the third level, indicated by colors from blue to heavy green, represents areas with the lowest concentrations.

The examination of the potassium radiation map (Fig. 11-A) reveals discernible patterns in potassium concentrations across various geological formations. The highest potassium values, representing the first level, are primarily associated with Calc-Alkaline Granitoids, Dukhan volcanic formations, and the Mut'iq group (comprising metasedimentary and metavolcanic rocks), with concentrations ranging from 1.6 to 3.5%. Intermediate potassium values, constituting the second level, are linked to Ophiolite and Calc-Alkaline volcanic groups, as well as Hammamat and Quaternary sediments, ranging from 0.26 to 1.6%. Conversely, the third zone, characterized by lower potassium concentrations below 0.26%, correlates closely with sedimentary formations such as the Nakhil, Thebes, Esna, Dakhla, and Duwwi formations.

The analysis of the equivalent uranium radiation map (Fig. 11-B) reveals three distinct concentration levels determined by uranium content. The first level, characterized by relatively higher concentrations, is primarily associated with Calc-Alkaline Granitoids, Mut'iq group, and formations such as Nakhil, Thebes, Esna, Dakhla, Duwwi, and Tarif, with values ranging from 4.56 to 12.8 ppm. Moderate uranium concentrations are identified in Dukhan volcanic, Hammamat sediments, and Quaternary deposits, ranging from 1.25 to 4.56 ppm. In contrast, Ophiolite and Calc-Alkaline volcanic groups exhibit lower uranium concentrations ranging from 0.2 to 1.25 ppm.

The equivalent thorium radiation map (Fig. 11-C) reveals three distinct levels of thorium concentrations across the study area. The highest level, ranging from 4.3 to 11.5 ppm, is primarily associated with Calc-Alkaline Granitoids, the Mut'iq group, Dakhla and Duwwi formations, Dukhan volcanic rocks, and Hammamat sediments. Intermediate thorium concentrations, ranging from 1.5 to 4.3 ppm, are observed in areas related to Calc-Alkaline volcanic and Ophiolite groups, as well as Quaternary deposits. The lowest level of thorium concentration, ranging from 0.5 to 1.5 ppm, is found in Evaporites, Nakhil, Thebes, and Esna formations, as well as certain parts of the Ophiolite group.

The distribution maps of radioelements serve as valuable tools for identifying specific rock types within the study area. For instance, the uranium map proves instrumental in delineating the boundaries of the Duwi sedimentary basin, offering precise insights into this geological formation. Conversely, the potassium and thorium maps offer valuable information regarding the distribution of igneous and metamorphic rocks. In the Gebel Duwi area, notably elevated uranium concentrations exceeding 12 ppm are observed over the Duwi formation, which comprises approximately 98 m of phosphatic limestone dating from the Santonian to Campanian age^71^. Additionally, there is a positive correlation between uranium content and phosphate concentration within the Duwi formation^72^.

##### Analysis of the radioelement ratios

**Figure 12:**
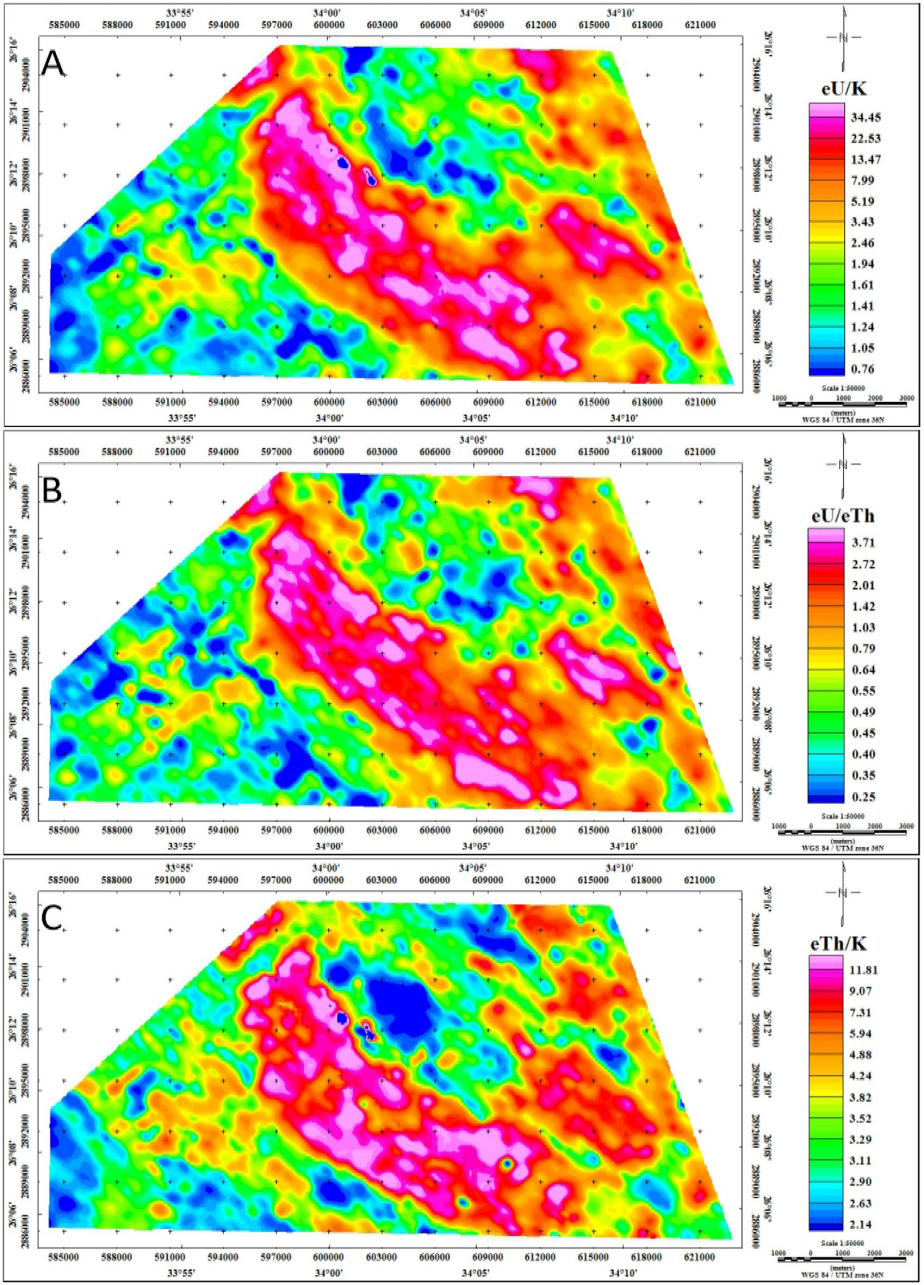
Radioelement ratios maps eU/K, eU/eTh, and eTh/K respectively of the Gebel Duwi area. (By Geosoft Oasis Montaj software https://www.seequent.com/products-solutions/geosoft-oasis-montaj/).

**Figure 13:**
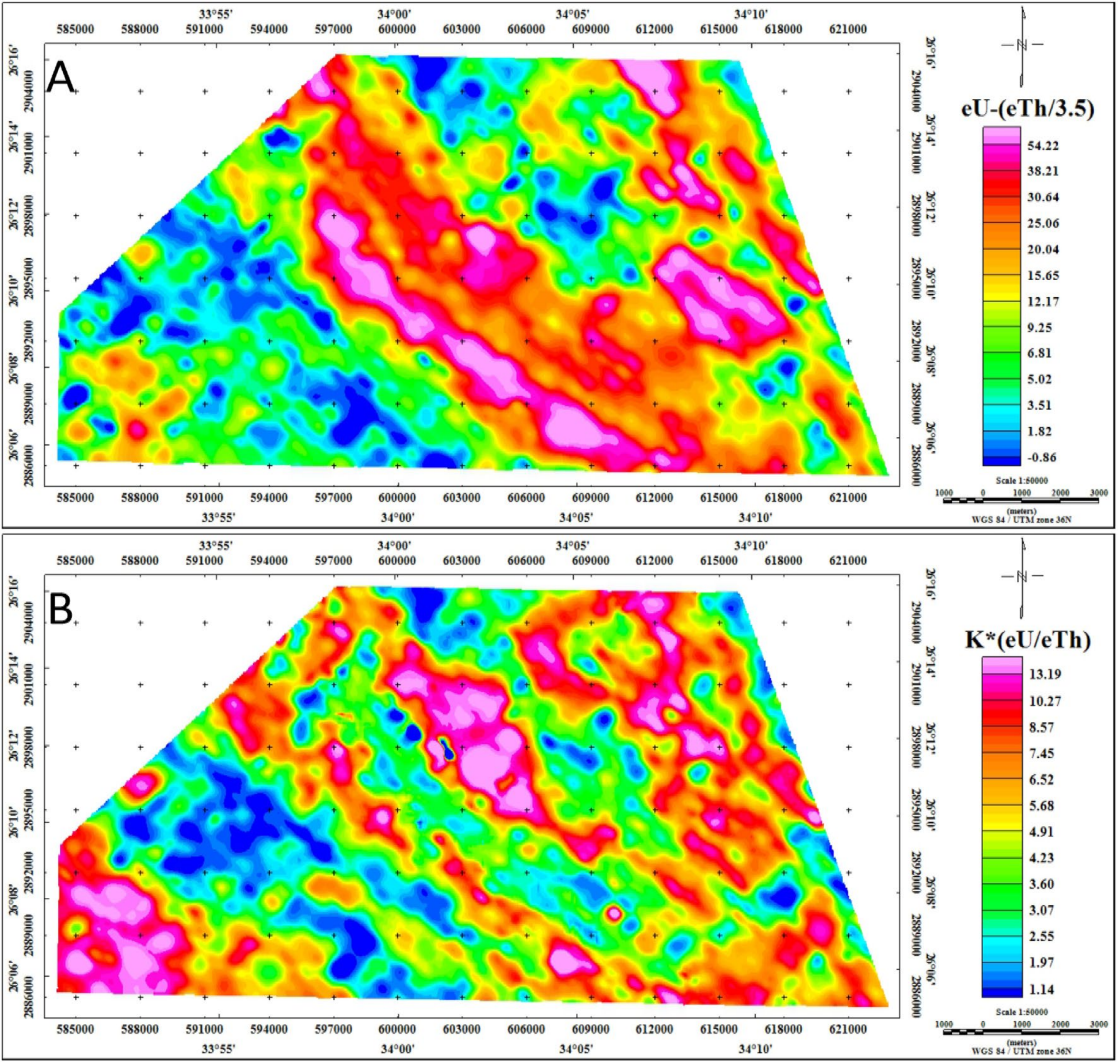
Uranium migration index and F-parameter maps of the Gebel Duwi area. (By Geosoft Oasis Montaj software https://www.seequent.com/products-solutions/geosoft-oasis-montaj/).

The utilization of radioelement ratios constitutes a cornerstone in mineral exploration, offering invaluable insights into the concentration interplay among distinct radioelements. Key ratios, including eU/K, eU/eTh, eTh/K, eU-(eTh/3.5), and K*(eU/eTh), hold significant importance in delineating mineralization zones (Figs. 12 and 13). Through the analysis of these ratios, scientists are empowered to unveil potential mineral deposits.

The eU/K ratio map proves to be a valuable asset in mineral exploration, effectively delineating areas with heightened uranium concentrations relative to potassium (Fig. 12-A). Particularly noteworthy are the regions where the eU/K ratio exceeds 12, found predominantly over sedimentary rocks in the central part of the study area. This includes formations such as Nakhil, Thebes, Esna, Dakhla, Duwwi, and Tarif, indicating a significant enrichment in uranium content. Moreover, Calc-Alkaline volcanic formations, Ophiolite groups, Dukhan volcanic rocks, and Hammamat sediments exhibit moderate concentrations of uranium in relation to potassium, ranging from 1.25 to 12. Conversely, Calc-Alkaline Granitoids and the Mut'iq group register eU/K ratio values below 1.25, suggesting a comparatively lower uranium concentration relative to potassium in these geological formations.

The eU/eTh ratio map provides a visual depiction of the relationship between equivalent uranium and equivalent thorium concentrations, offering valuable insights into uranium enrichment patterns (Fig. 12-B). Notably, the uranium enrichment observed on the eU/eTh ratio map corresponds closely with the periphery of the potassium anomaly, a phenomenon previously documented^73^. The most prominent eU/eTh ratio anomaly, surpassing 1.5, is intricately associated with the strongly altered mineralized zone within the sedimentary rocks, including formations such as Nakhil, Thebes, Esna, Dakhla, Duwwi, and Tarif, within the study area.

The eTh/K ratio map emerges as a powerful tool for identifying mineralization, offering a nuanced perspective by assessing the ratio of equivalent thorium to potassium concentrations (Fig. 12-C). Elevated eTh/K ratios serve as key indicators of potential mineralization zones, enabling a focused approach to pinpoint areas of heightened geological importance^26^. Particularly noteworthy are the Nakhil, Thebes, Esna, Dakhla, Duwwi, and Tarif formations, which exhibit the highest eTh/K ratio values, surpassing 7, highlighting a substantial concentration of thorium relative to potassium within these geological formations. Additionally, Dukhan volcanics, Hammamat sediments, Calc-Alkaline volcanic, and Ophiolite groups demonstrate intermediate eTh/K ratio values, indicating a moderate thorium concentration in relation to potassium. Conversely, certain portions of Calc-Alkaline Granitoids and the Mut'iq group register eTh/K ratio values below 3.11, suggesting a relatively lower thorium concentration in these specific areas.

The uranium migration index, also known as the original equivalent uranium concentration (OUC) map, was calculated to investigate the movement of uranium within various geological zones^63,74^. This analysis is complemented by the eU-(eTh/3.5) mobilization map, which highlights a significant mobilization phase of uranium into mineralized zones (Fig. 13-A). This phenomenon indicates the substantial migration of uranium as altered rocks interact with mineralization-bearing solutions, leading to the enrichment of uranium mineralization. The resulting map provides a comparative analysis between the original uranium distribution and its updated counterpart in the study area. It is noteworthy that there was a depletion of uranium, primarily observed around the center of the study area, characterized by negative values of uranium. Conversely, regions trending from northwest to southeast, particularly along the center of the study area, exhibit uranium deposition, as evidenced by positive values of the index. These areas predominantly include sedimentary rocks in the central part of the study area, notably the Nakhil, Thebes, Esna, Dakhla, Duwwi, and Tarif formations.

The identification of mineralized alteration zones, as outlined^75^, involves a normalization technique calculated using the F parameter, a valuable metric proven effective in delineating potassic alterations associated with mineralization, with values exceeding 10 for altered rocks^15,26^. In the Gebel Duwi area, the Ophiolite group and Evaporites register below 4.91, which is considered the lowest level. Hammamat sediments, Calc-Alkaline volcanic, and the Nakhil, Thebes, Esna, Dakhla, Duwwi, and Tarif formations fall within the range of 4.91 to 10, indicating an intermediate level. Notably, specific areas within Calc-Alkaline Granitoids, the Mut'iq group, Dukhan volcanic, and certain portions of sedimentary formations exhibit the highest F-parameter values, exceeding 10 (Fig. 13-B). By delineating areas showcasing elevated F-parameter anomaly values, this approach effectively highlights zones conducive to uranium mineralization.

#### Quantitative interpretation

##### Statistical treatment of the aero-spectrometric data

The quantitative interpretation of the aero-spectrometric survey data focuses on identifying surface radioactive anomalies^16^. This interpretation relies on the understanding that the absolute and relative concentrations of radioelements (K, eU, and eTh) vary significantly depending on the lithology^13^. This approach involves statistical analysis of the original spectrometric data for potassium, equivalent uranium, and equivalent thorium without applying any transformations.

**Figure 14:**
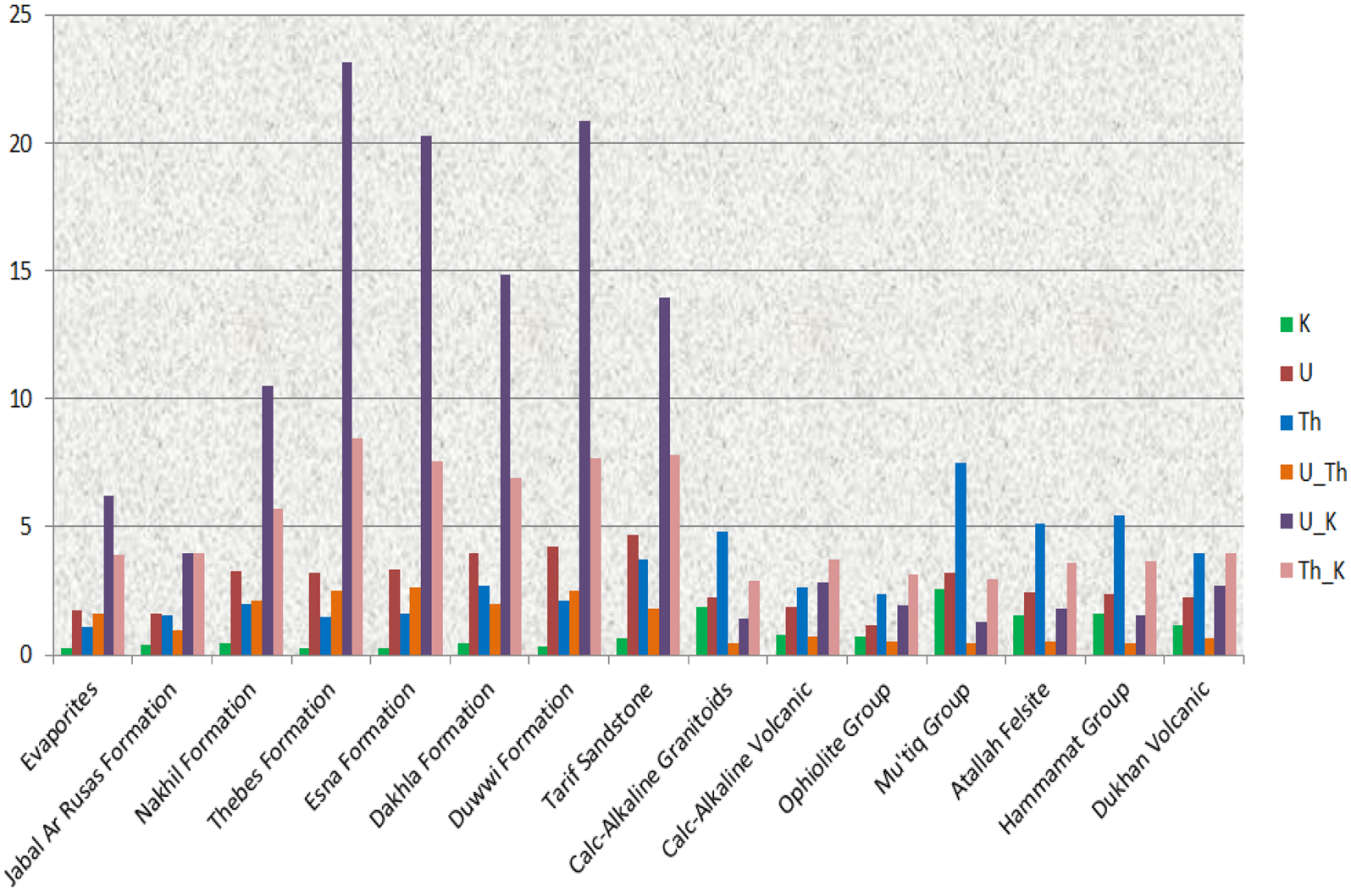
Frequencies histograms of mean values of the aero-spectrometric variables and their correspondence rock units.

The airborne gamma-ray spectrometric data, including measurements of three radiometric variables (K, eU, and eTh), along with their ratios (eU/K, eU/eTh, and eTh/K), were subjected to statistical analysis to understand the distribution and significance of these radioelements across the study area, as shown in Table 3. Furthermore, the normality and homogeneity of the distributions of radioelements were assessed by calculating the coefficient of variability (C.V. = S/X * 100) for each radiometric parameter across various rock types^76^. Additionally, frequency histograms were constructed to visualize the total radioactivity of significant radioactive rock units. The arithmetic mean (X) values of the variables, as well as their corresponding rock units, are presented in Fig. 14.

**Table 3:** Statistical parameters of gamma-ray spectrometric radioelements within the rock units of the Gebel Duwi area.

Rock units	Statistical parameters	K (%)	eU (ppm)	eTh (ppm)	eU/eTh	eU/K	eTh/K
Evaporites	Max	0.411	5.725	1.859	5.671	19.014	5.336
	Min	0.166	0.383	0.65	0.383	1.237	2.446
	X	0.276	1.735	1.06	1.586	6.228	3.898
	S	0.065	1.307	0.259	1.143	4.251	0.66
	C.V.	23.409	75.304	24.386	72.087	68.267	16.934
	X+1S	0.34	3.042	1.318	2.729	10.479	4.558
	X+2S	0.405	4.349	1.577	3.872	14.73	5.218
	X+3S	0.469	5.655	1.835	5.015	18.982	5.878
	X−1S	0.211	0.429	0.801	0.443	1.976	3.238
	X−2S	0.147	− 0.878	0.543	− 0.701	− 2.275	2.578
	X−3S	0.082	− 2.185	0.284	− 1.844	− 6.527	1.918
Jabal Ar Rusas	Max	0.651	3.624	2.283	1.588	6.763	5.202
	Min	0.179	0.449	0.884	0.457	1.662	2.626
	X	0.406	1.614	1.526	0.992	3.941	3.960
	S	0.148	0.88	0.386	0.354	1.667	0.724
	C.V.	36.311	54.523	25.3	35.718	42.31	18.272
	X+1S	0.554	2.493	1.912	1.346	5.609	4.684
	X+2S	0.702	3.373	2.298	1.701	7.276	5.407
	X+3S	0.85	4.253	2.685	2.055	8.944	6.131
	X−1S	0.259	0.734	1.14	0.638	2.274	3.237
	X−2S	0.111	− 0.146	0.754	0.283	0.606	2.513
	X−3S	− 0.036	− 1.026	0.368	− 0.071	− 1.061	1.789
Nakhil formation	Max	3.231	10.714	6.955	18.276	201.575	62.986
	Min	− 0.066	0.871	0.233	0.37	− 2051.324	− 425.357
	X	0.457	3.257	1.97	2.111	10.485	5.718
	S	0.486	1.123	1.045	1.549	90.62	19.303
	C.V.	106.457	34.469	53.07	73.376	864.256	337.57
	X+1S	0.943	4.379	3.015	3.66	101.106	25.021
	X+2S	1.429	5.502	4.06	5.209	191.726	44.323
	X+3S	1.916	6.625	5.106	6.758	282.346	63.626
	X−1S	− 0.029	2.134	0.924	0.562	− 80.135	− 13.585
	X−2S	− 0.516	1.012	− 0.121	− 0.987	− 170.755	− 32.887
	X−3S	− 1.002	− 0.111	− 1.166	− 2.536	− 261.376	− 52.19
Thebes formation	Max	1.482	9.746	4.949	11.899	179.467	45.322
	Min	− 0.1	0.544	0.337	0.319	− 0.002	− 0.001
	X	0.253	3.21	1.495	2.524	23.186	8.468
	S	0.261	1.239	0.704	1.357	16.881	4.241
	C.V.	102.948	38.593	47.07	53.759	72.805	50.087
	X+1S	0.514	4.449	2.198	3.88	40.067	12.709
	X+2S	0.775	5.688	2.902	5.237	56.948	16.951
	X+3S	1.035	6.926	3.605	6.594	73.829	21.192
	X−1S	− 0.007	1.971	0.791	1.167	6.306	4.227
	X−2S	− 0.268	0.732	0.088	− 0.19	− 10.575	− 0.015
	X−3S	− 0.529	− 0.506	− 0.616	− 1.546	− 27.456	− 4.256
Esna formation	Max	1.489	7.594	5.571	11.915	74.92	18.812
	Min	0.06	1.531	0.377	0.45	1.292	2.77
	X	0.279	3.299	1.583	2.631	20.267	7.564
	S	0.299	1.385	0.98	1.695	12.846	3.01
	C.V.	107.577	41.986	61.857	64.441	63.385	39.8
	X+1S	0.579	4.683	2.563	4.326	33.113	10.574
	X+2S	0.879	6.068	3.543	6.021	45.959	13.585
	X+3S	1.179	7.453	4.523	7.716	58.805	16.595
	X−1S	− 0.021	1.9136	0.604	0.935	7.421	4.553
	X−2S	− 0.321	0.529	− 0.376	− 0.76	− 5.425	1.543
	X−3S	− 0.621	− 0.856	− 1.356	− 2.455	− 18.272	− 1.467
Dakhla formation	Max	1.582	10.554	8.17	11.929	92.323	22.947
	Min	0.044	1.094	0.337	0.338	1.137	2.723
	X	0.455	3.984	2.695	1.993	14.873	6.886
	S	0.315	1.999	1.611	1.379	13.053	2.796
	C.V.	69.262	50.199	59.796	69.181	87.763	40.605
	X+1S	0.77	5.984	4.306	3.372	27.926	9.682
	X+2S	1.085	7.984	5.917	4.751	40.978	12.478
	X+3S	1.4	9.983	7.529	6.13	54.031	15.275
	X−1S	0.14	1.984	1.083	0.614	1.82	4.09
	X−2S	− 0.175	− 0.016	− 0.528	− 0.765	− 11.233	1.294
	X−3S	− 0.49	− 2.016	− 2.139	− 2.144	− 24.286	− 1.502
Duwwi formation	Max	1.481	9.005	6.052	6.59	72.933	26.168
	Min	0.068	0.808	0.5	0.226	0.915	2.49
	X	0.333	4.202	2.091	2.525	20.851	7.699
	S	0.265	1.955	1.096	1.406	15.138	3.507
	C.V.	79.547	46.523	52.378	55.675	72.601	45.549
	X+1S	0.598	6.157	3.186	3.931	35.99	11.206
	X+2S	0.863	8.111	4.281	5.337	51.128	14.713
	X+3S	1.129	10.066	5.376	6.743	66.267	18.22
	X−1S	0.068	2.247	0.996	1.119	5.713	4.192
	X−2S	− 0.197	0.292	− 0.099	− 0.287	− 9.425	0.685
	X−3S	− 0.462	− 1.663	− 1.195	− 1.693	− 24.564	− 2.822
Tarif sandstone	Max	2.915	12.825	8.59	12.451	73.528	26.904
	Min	0.087	0.996	0.537	0.225	0.884	2.073
	X	0.623	4.667	3.691	1.782	13.979	7.838
	S	0.516	2.102	1.583	1.599	12.873	3.784
	C.V.	82.805	45.026	42.903	89.742	92.091	48.284
	X+1S	1.14	6.769	5.274	3.381	26.851	11.622
	X+2S	1.656	8.87	6.857	4.98	39.725	15.406
	X+3S	2.172	10.972	8.441	6.578	52.598	19.191
	X−1S	0.107	2.566	2.107	0.183	1.106	4.053
	X−2S	− 0.409	0.464	0.524	− 1.416	− 11.767	0.269
	X−3S	− 0.925	− 1.637	− 1.06	− 3.015	− 24.641	− 3.515
Calc-alkaline granitoids	Max	3.502	7.922	8.274	1.649	6.837	8.951
	Min	− 0.1	0.281	− 0.1	− 0.002	− 0.002	1
	X	1.862	2.239	4.79	0.478	1.387	2.886
	S	0.867	0.907	1.541	0.194	0.832	1.058
	C.V.	46.584	40.496	32.18	40.545	60.013	36.678
	X+1S	2.729	3.145	6.331	0.671	2.219	3.944
	X+2S	3.596	4.052	7.873	0.865	3.052	5.003
	X+3S	4.464	4.959	9.414	1.059	3.884	6.061
	X−1S	0.995	1.332	3.248	0.284	0.555	1.827
	X−2S	0.127	0.426	1.707	0.09	− 0.278	0.769
	X−3S	− 0.74	− 0.481	0.166	− 0.103	− 1.11	− 0.29
Calc-alkaline volcanic group	Max	2.192	8.857	7.291	4.681	27.318	15.111
	Min	0.202	0.161	0.935	0.077	0.288	1.476
	X	0.758	1.837	2.628	0.712	2.81	3.687
	S	0.288	1.435	1.028	0.553	3.237	1.581
	C.V.	37.987	78.101	39.102	77.607	115.199	42.884
	X+1S	1.046	3.271	3.656	1.265	6.046	5.268
	X+2S	1.334	4.706	4.683	1.817	9.283	6.849
	X+3S	1.623	6.14	5.711	2.37	12.52	8.43
	X−1S	0.47	0.402	1.6	0.159	− 0.427	2.106
	X−2S	0.182	− 1.032	0.573	− 0.393	− 3.664	0.525
	X−3S	− 0.106	− 2.467	− 0.455	− 0.946	− 6.9	− 1.056
Ophiolite group	Max	2.808	4.898	8.843	2.033	1.75E + 01	1.74E + 01
	Min	− 0.1	0.111	− 0.1	− 0.0005	− 1.93E− 03	− 8.82E+02
	X	0.699	1.156	2.401	0.489	1.93E+00	3.16E+00
	S	0.409	0.752	1.165	0.208	1.670	23.701
	C.V.	58.551	65.011	48.525	42.658	86.586	749.468
	X+1S	1.108	1.908	3.566	0.697	3.599	26.864
	X+2S	1.517	2.659	4.731	0.906	5.27	50.565
	X+3S	1.926	3.411	5.897	1.114	6.94	74.267
	X−1S	0.29	0.405	1.236	0.28	0.259	− 20.539
	X−2S	− 0.119	− 0.347	0.071	0.072	− 1.412	− 44.24
	X−3S	− 0.529	− 1.099	− 1.094	− 0.137	− 3.082	− 67.942
Mu’tiq group	Max	3.393	7.287	11.532	1	3.729	5.471
	Min	− 0.1	− 0.1	− 0.1	0.15	0.417	1
	X	2.557	3.216	7.483	0.436	1.309	2.977
	S	0.682	1.194	2.096	0.113	0.451	0.54
	C.V.	26.678	37.144	28.014	25.821	34.454	18.143
	X+1S	3.24	4.41	9.579	0.549	1.76	3.517
	X+2S	3.922	5.604	11.676	0.661	2.21	4.057
	X+3S	4.604	6.799	13.772	0.774	2.661	4.597
	X−1S	1.875	2.021	5.387	0.323	0.858	2.437
	X−2S	1.193	0.827	3.29	0.211	0.407	1.897
	X−3S	0.511	− 0.368	1.194	0.098	− 0.044	1.357
Atallah Felsite	Max	2.488	3.775	6.815	0.954	5.641	7.271
	Min	0.554	1.268	2.884	0.274	1.08	2.699
	X	1.526	2.465	5.139	0.484	1.771	3.571
	S	0.472	0.637	0.93	0.123	0.879	0.823
	C.V.	30.902	25.842	18.089	25.472	49.63	23.048
	X+1S	1.998	3.103	6.068	0.607	2.651	4.394
	X+2S	2.469	3.74	6.998	0.731	3.53	5.217
	X+3S	2.941	4.377	7.928	0.854	4.409	6.04
	X−1S	1.054	1.828	4.209	0.361	0.892	2.748
	X−2S	0.583	1.191	3.28	0.237	0.013	1.925
	X−3S	0.111	0.554	2.35	0.114	− 0.866	1.102
Hammamat group	Max	2.675	4.151	8.201	0.592	3.073	8.251
	Min	0.522	0.814	2.21	0.2	1.027	2.631
	X	1.595	2.37	5.449	0.432	1.526	3.644
	S	0.573	0.837	1.408	0.088	0.325	0.952
	C.V.	35.923	35.308	25.845	20.334	21.299	26.124
	X+1S	2.168	3.207	6.857	0.52	1.851	4.596
	X+2S	2.741	4.043	8.265	0.607	2.176	5.548
	X+3S	3.314	4.88	9.674	0.695	2.501	6.501
	X−1S	1.022	1.53	4.04	0.344	1.201	2.692
	X−2S	0.449	0.697	2.632	0.256	0.876	1.74
	X−3S	− 0.124	− 0.14	1.224	0.168	0.551	0.788
Dukhan volcanic	Max	2.572	5.715	8.198	3.685	18.843	16.459
	Min	− 0.1	0.25	0.384	0.113	− 0.001	− 0.001
	X	1.125	2.272	3.945	0.645	2.713	3.973
	S	0.533	0.969	1.425	0.415	2.65	1.736
	C.V.	47.353	42.645	36.123	64.309	97.684	43.705
	X+1S	1.658	3.24	5.37	1.059	5.364	5.709
	X+2S	2.191	4.209	6.795	1.474	8.014	7.445
	X+3S	2.724	5.178	8.22	1.888	10.664	9.181
	X−1S	0.593	1.303	2.52	0.23	0.063	2.236
	X−2S	0.06	0.334	1.09	− 0.184	− 2.588	0.5
	X−3S	− 0.473	− 0.635	− 0.33	− 0.599	− 5.238	− 1.236

The careful examination of the statistical parameters of gamma-ray spectrometric radioelements reveals notable trends in the mean concentrations across different rock formations. Particularly, the Tarif and Duwwi formations exhibit the highest mean equivalent uranium concentrations, measuring at 4.667 and 4.202 ppm, respectively. Additionally, the Mut'iq group and Calc-Alkaline Granitoids are associated with the highest mean potassium concentrations, recorded at 2.557% and 1.862%, respectively. Moreover, elevated mean equivalent thorium concentrations are observed in the Mut'iq group and Hammamat sediments, with values reaching 7.483 and 5.449 ppm, respectively. Furthermore, the Duwwi formation dominates in mean ratio values of eU, eU/eTh, and eU/K in the study area, recording values of 4.202, 2.525, and 20.851, respectively. Additionally, the Thebes formation exhibits the highest mean ratio values of eU/K and eTh/K, at 23.186 and 8.468, respectively. Finally, in terms of the mean eU/eTh ratio value, the Esna formation demonstrates the highest value, reaching 2.631 across the study area.

##### Outlining the uranium provinces zones

The primary aim of interpreting airborne gamma-ray spectrometric survey data is to delineate potential uranium-rich regions characterized by elevated uranium concentrations in rocks and soils^51^. Subsequently, upon identifying these target areas, more comprehensive ground prospecting techniques can be employed to assess their economic viability. From an exploration standpoint, key parameters include the relative concentrations of uranium to potassium and uranium to thorium, particularly when analyzed alongside uranium measurements. These parameters serve as diagnostic indicators for detecting zones with anomalous uranium concentrations^50^.

**Figure 15:**
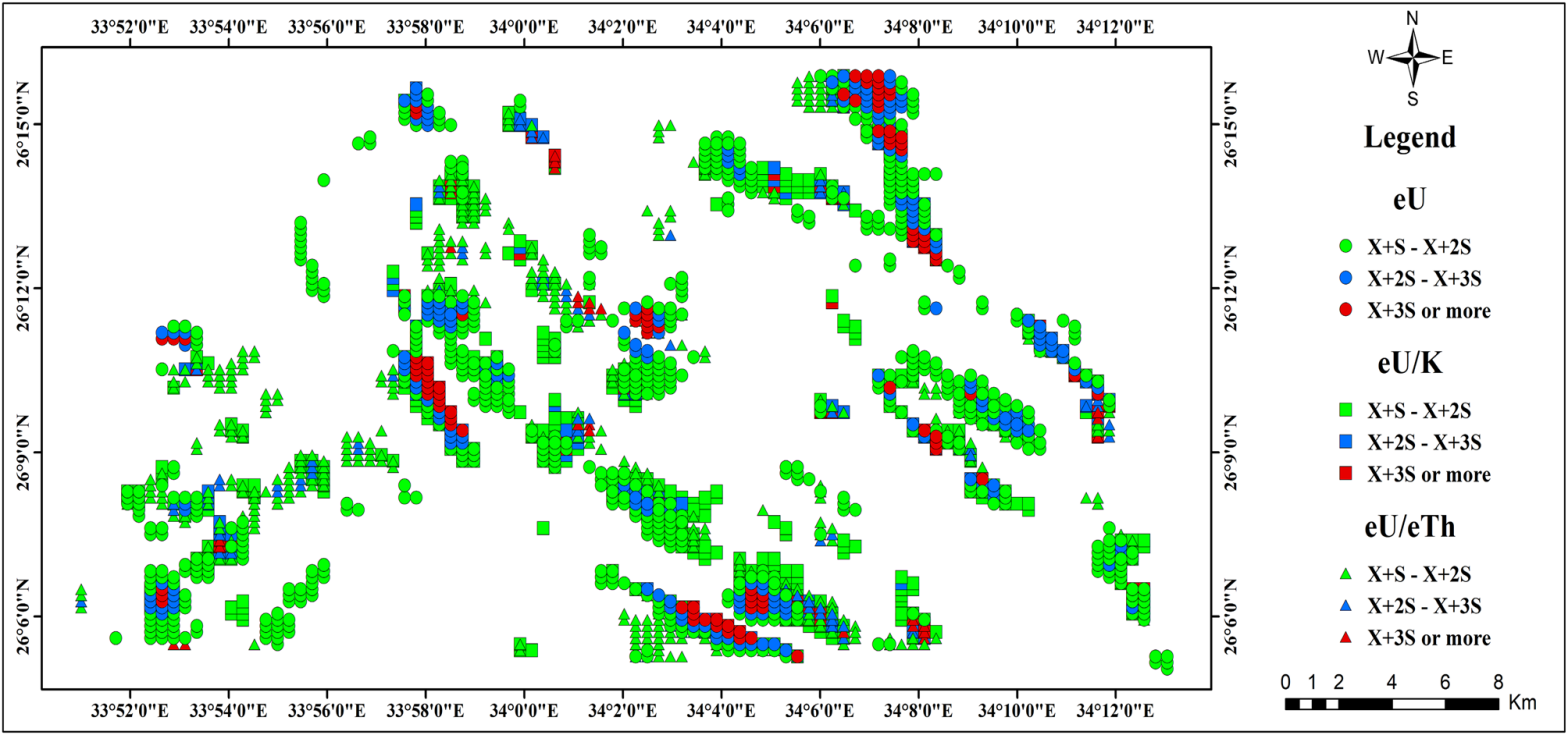
The uranium point anomaly map of the Gebel Duwi area. (By ArcGIS v.10.5. https://www.esri.com/en-us/arcgis/products/arcgis-desktop/overview/).

**Figure 16:**
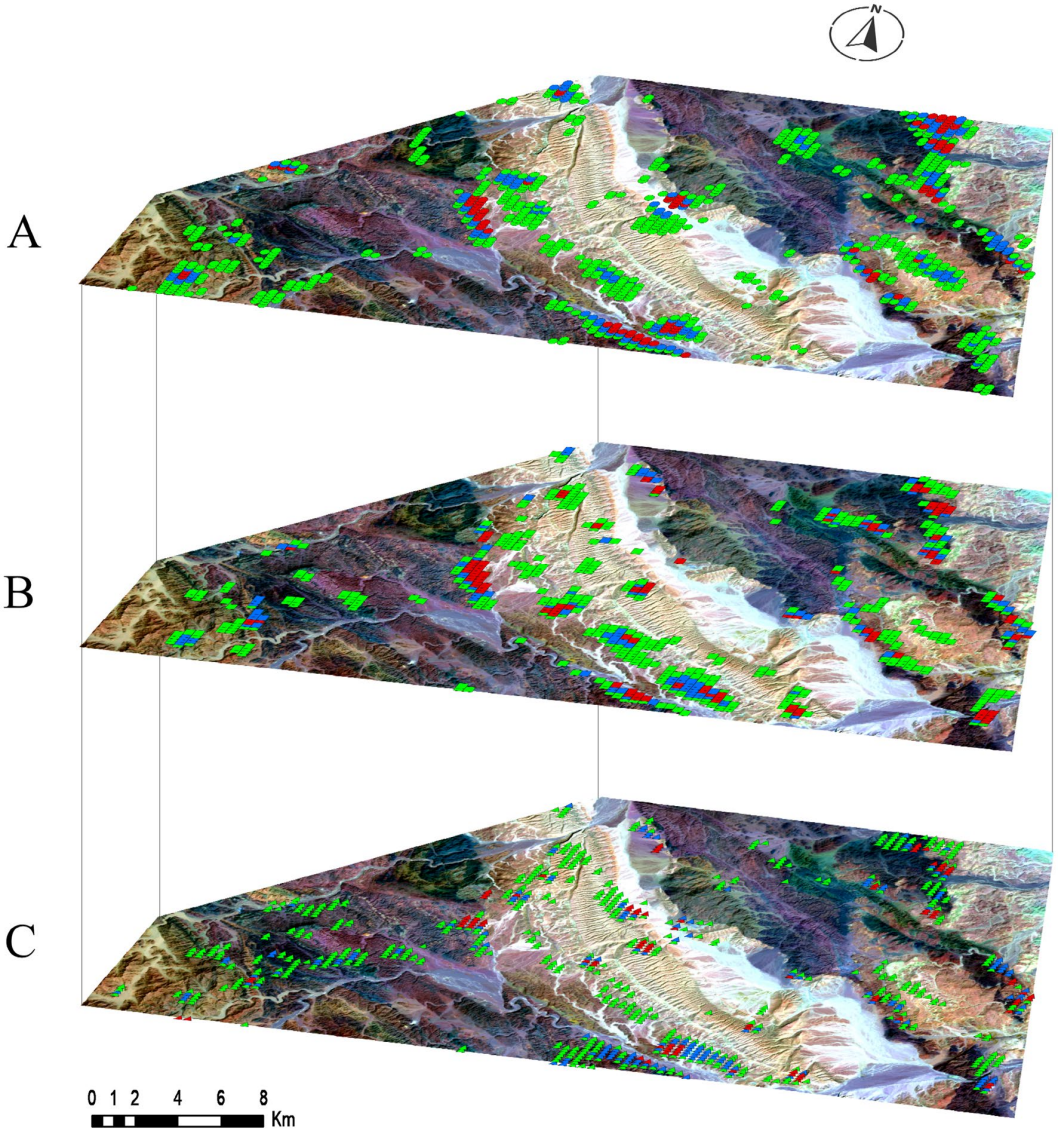
The uranium point anomaly map, comparison of layers (**A**) eU, (**B**) eU/K, and (**C**) eU/eTh variables in the Gebel Duwi area. (By ArcGIS v.10.5. https://www.esri.com/en-us/arcgis/products/arcgis-desktop/overview/).

These zones can be identified through a probabilistic approach based on the deviation of the data from the mean background, as defined by the dataset itself. These deviations are quantified in terms of specific levels of probability. The threshold for determining high anomalous values is set at two or three standard deviations above the calculated arithmetic mean value (X + 2S or 3S) for each rock unit, as outlined in Table 3, across eU, eU/eTh, and eU/K measurements. This established threshold serves as a reliable criterion for distinguishing between normal and abnormal variables, helping to identify anomalous zones^51^. The application of this methodology is depicted through the uranium point anomaly map (Fig. 15), which highlights areas surpassing the thresholds of (X + 2S) and (X + 3S) for the eU, eU/K, and eU/Th variables. Additionally, (Fig. 16) provides a comparative analysis of these variables across the study area, designated as (A), (B), and (C) for eU, eU/K, and eU/eTh respectively. A careful analysis of this map reveals that uranium province zones are associated with specific locations within the Calc-Alkaline Granitoids, the Mut'iq and Ophiolite groups, as well as the sedimentary formations, including Nakhil, Thebes, Esna, Dakhla, Duwwi, and Tarif formations. This association is evidenced by values exceeding (X + 2S) and (X + 3S) for eU, eU/K, and eU/eTh.

### Field study and mineralogical analysis

**Figure 17:**
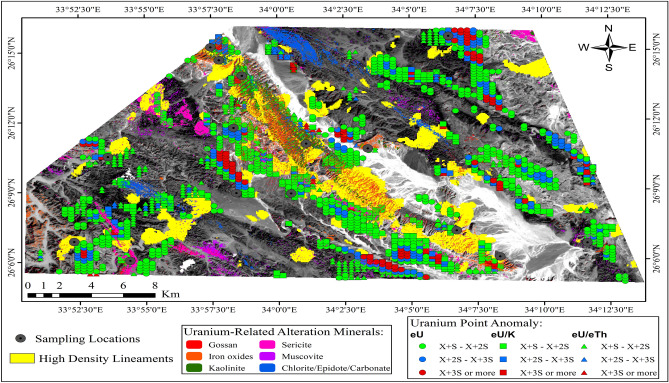
Comprehensive integration of uranium-alteration minerals identified through remote sensing techniques, uranium point anomaly locations from airborne geophysics, and high-density lineaments, highlighting uranium potential zones. (By ArcGIS v.10.5. https://www.esri.com/en-us/arcgis/products/arcgis-desktop/overview/).

**Figure 18:**
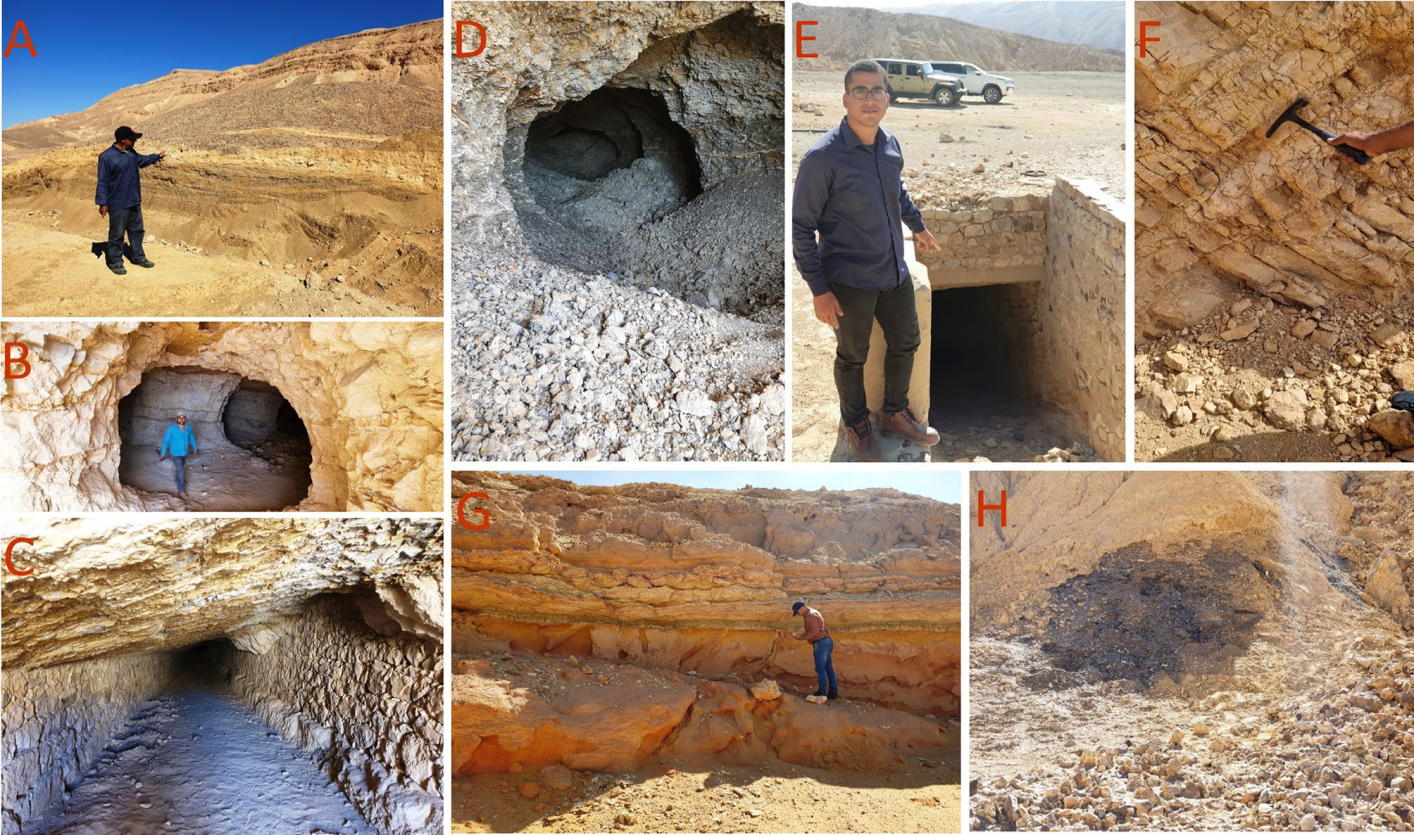
Signs of highly potential localities for uranium mineralization observed during the field investigation; (**A**) alternations of phosphate beds associating with oil shale bed, (**B**) tunnel in the phosphate beds, (**C**) long tunnel in the limestone hosting phosphate, (**D**) tunnel in the phosphate beds associating with oil shale, (**E**) supported tunnel in the phosphate beds, (**F**) alternations of phosphate beds in the limestone host rocks, (**G**) repetitions of inter-bedded phosphate beds in the limestone, and (**H**) associations of phosphate beds and patches of oil shale bed in the Duwi area. (The person shown in the image is the author- Mahmoud Abd El-Rahman Hegab).

Initially, we commenced by identifying uranium-related hydrothermal alteration minerals and meticulously mapped their spatial distribution. Subsequently, we correlated these findings with areas characterized by high-density lineaments, serving as prominent indicators of underlying geological structures associated with mineralization. Further enhancing our analysis, we meticulously examined gamma-ray spectrometric data to pinpoint zones displaying elevated uranium anomalies. This comprehensive approach culminated in the precise delineation of uranium-rich zones within the study area, as illustrated in Fig. 17. To authenticate our findings, we embarked on a comprehensive field study, during which we meticulously collected seventeen rock samples from the identified uranium mineralized locations, as presented in Fig. 18. Each sample was thoughtfully selected to encapsulate the diverse range of geological formations present within the uranium-rich zones. Leveraging cutting-edge technology, we employed the ASD TerraSpec Halo Mineral Identifier Device to scrutinize the mineral composition of these samples, leveraging their distinctive spectral signatures for precise identification and analysis as illustrated in Table 4.

**Table 4:** Characteristics of the selected rock samples from the uranium-rich zones in the Gebel Duwi area.

Sample no.	Identified minerals	Sample image	Spectral profile
D-1	Fe-Chlorite, Ankerite, Biotite, Gypsum		
D-2	Ferrihydrite		
D-3	Laumontite, Calcite		
D-4	Goethite, Harmotome, Dolomite		
D-5	Chabazite, Calcite, Hornblende		
D-6	Fe-Mg-Chlorite, Clinozoisite		
D-7	Ferrihydrite, Fe-Mg-Chlorite, Muscovite		
D-8	Fe-Mg-Chlorite, Vermiculite, Gypsum, Muscovite		
D-9	Goethite, Antigorite, Brucite		
D-10	Ferrihydrite, Actinolite, Fe-Mg-Chlorite, Brucite		
D-11	Muscovite, Fe-Mg-Chlorite		
D-12	Ferrihydrite, Phengite, Fe-Mg-Chlorite		
D-13	Ferrihydrite, Phengite		
D-14	Ferrihydrite, Harmotome, Calcite, K-illite, Dolomite, Goethite		
D-15	Vermiculite, Muscovite, Fe-Chlorite		
D-16	Ferrihydrite, Muscovite, Vermiculite, Ankerite, Gypsum, Goethite		
D-17	Ferrihydrite, Antigorite		

Hydrothermal alteration zones, such as iron oxides, argillic, propylitic, and phyllic alterations, are frequently observed in close proximity to uranium deposits^11,12,77^. After analyzing the selected rock samples, we concluded that the identified minerals have a significant association with uranium mineralization. Fe-Chlorite, found in samples D-1 and D-15, can form during the alteration of primary uranium-bearing minerals. Fe-Mg-Chlorite, identified in samples D-6, D-7, D-8, D-10, D-11, and D-12, is commonly associated with uranium deposits and can serve as an indicator of hydrothermal alteration zones related to uranium mineralization. Chlorite stands out as a key hydrothermal mineral found in numerous uranium deposits worldwide^78^. Samples D-2, D-7, D-10, D-12, D-13, D-14, D-16, and D-17 containing ferrihydrite suggest a potential association with uranium mineralization processes, as ferrihydrite can form during the oxidative weathering of primary uranium-bearing minerals. Samples D-4, D-9, D-14, and D-16 containing goethite suggest a possible connection to uranium mineralization, as goethite is often associated with the weathering and alteration of primary uranium minerals. Iron oxides, hydroxides, and sulfides are particularly significant as they can interact with uranium-bearing fluids and promote the formation of uranium minerals^77^. Additionally, the presence of calcite in samples D-3, D-5, and D-14 indicates potential uranium mineralization, as calcite can precipitate from hydrothermal fluids carrying uranium^79^. Muscovite, present in samples D-7, D-8, D-11, D-15, and D-16, is commonly found in uranium deposits, suggesting potential areas of interest for uranium exploration^11^. Samples D-4 and D-14 containing dolomite may occur as gangue minerals in certain types of uranium deposits^79^. Sample D-10 containing actinolite and sample D-15 containing vermiculite further highlight potential associations with uranium mineralization processes^80^. Moreover, samples D-1, D-8, and D-14 containing gypsum may act as pathways for uranium-bearing fluids to migrate and precipitate uranium minerals^81^.

**Figure 19:**
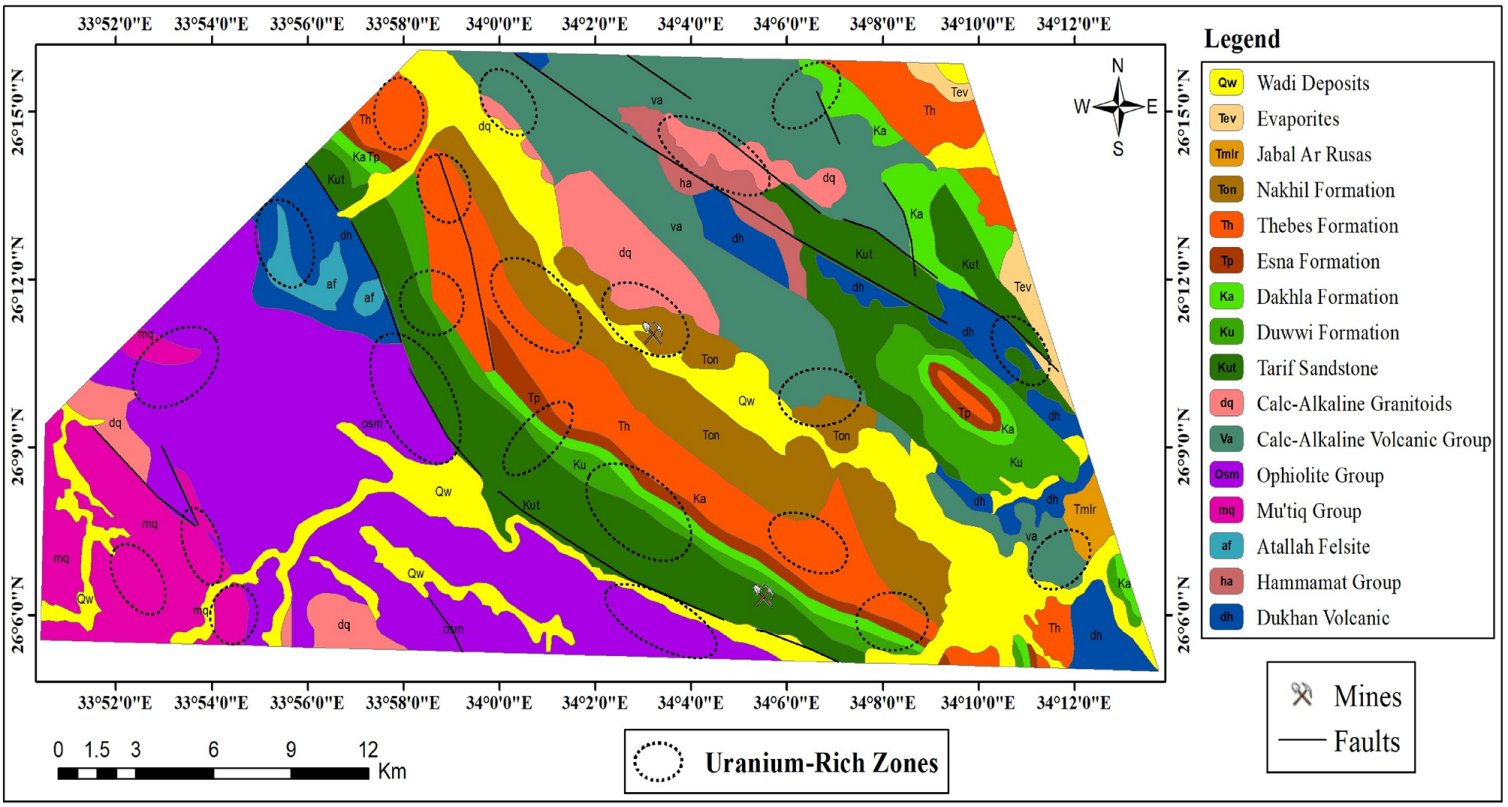
The uranium potential mineralization zones on the geological background of the Gebel Duwi area. (By ArcGIS v.10.5. https://www.esri.com/en-us/arcgis/products/arcgis-desktop/overview/).

Through the integration of remote sensing and airborne gamma-ray spectrometry data, we generated a uranium potential map, pinpointing areas with elevated concentrations indicative of uranium mineralization (Fig. 19). This methodology and the resultant findings were validated through field studies and mineralogical analyses of collected samples. The analysis revealed that samples obtained from areas identified as having high uranium potential contained minerals indicative of uranium alteration. These results underscore the importance of further exploration and investigation in the study area, emphasizing its potential for significant uranium mineralization.

## Conclusion

This study provides a comprehensive analysis of uranium potential in the Gebel Duwi area in the Central Eastern Desert of Egypt by employing a synergistic approach that integrates remote sensing and airborne gamma-ray spectrometric data analyses. Utilizing multispectral remote sensing techniques, alongside airborne gamma-ray spectrometry data, the study identifies hydrothermal alteration zones associated with uranium deposition. Additionally, the spatial distribution of radioelements, including uranium, thorium, and potassium, and their ratios, is thoroughly examined, further enhancing our understanding of uranium mineralization. Statistical analyses, including mean, standard deviation, and coefficient of variability, aid in identifying uranium-rich zones. Through the integration of these datasets, a uranium potential map is generated, highlighting areas with elevated concentrations indicative of uranium mineralization. Through field study and mineralogical analyses of the collected samples, we have validated our findings, confirming the presence of minerals intimately associated with uranium mineralization. Specifically, our investigations have identified the presence of key minerals such as Fe-Chlorite, Fe-Mg-Chlorite, ferrihydrite, goethite, calcite, muscovite, dolomite, actinolite, vermiculite, and gypsum within the delineated high-potential areas mapped during our study. Thus, the adopted methodology is recommended to be followed for uranium mineralization investigations.

## Data Availability

Data sets generated during the current study are available from the corresponding author on reasonable request.
